# Direct measurements of mRNA translation kinetics in living cells

**DOI:** 10.1038/s41467-022-29515-x

**Published:** 2022-04-06

**Authors:** Mikhail Metelev, Erik Lundin, Ivan L. Volkov, Arvid H. Gynnå, Johan Elf, Magnus Johansson

**Affiliations:** Department of Cell and Molecular Biology, Uppsala University, Uppsala, Sweden

**Keywords:** Ribosome, Single-molecule biophysics

## Abstract

Ribosome mediated mRNA translation is central to life. The cycle of translation, however, has been characterized mostly using reconstituted systems, with only few techniques applicable for studies in the living cell. Here we describe a live-cell ribosome-labeling method, which allows us to characterize the whole processes of finding and translating an mRNA, using single-molecule tracking techniques. We find that more than 90% of both bacterial ribosomal subunits are engaged in translation at any particular time, and that the 30S and 50S ribosomal subunits spend the same average time bound to an mRNA, revealing that 30S re-initiation on poly-cistronic mRNAs is not prevalent in *E. coli*. Instead, our results are best explained by substantial 70S re-initiation of translation of poly-cistronic mRNAs, which is further corroborated by experiments with translation initiation inhibitors. Finally, we find that a variety of previously described orthogonal ribosomes, with altered anti-Shine-Dalgarno sequences, show significant binding to endogenous mRNAs.

## Abstract (web summary)

Metelev et al. use single-molecule tracking to study kinetics of translation directly in *E. coli* cells, and how it is affected by translation inhibitors and rRNA mutations. Their results support widespread 70S re-initiation on mRNAs.

## Introduction

Translation of mRNAs into proteins is one of the most fundamental molecular processes in all living organisms. Central to this process is the ribosome, a highly conserved macromolecular machine consisting of two subunits, the small (30S in bacteria), and the large (50S in bacteria). mRNA translation has been extensively studied over the decades, and a wide variety of bulk biochemical approaches and structural techniques have helped paint a detailed picture of the process^1^. Recent advances in cryogenic electron microscopy continue to provide new snapshots of ribosomes bound to ligands, capturing multiple conformations, and revealing previously hidden structural dynamics^2,3^. Emerging single-molecule techniques have, at the same time, helped to connect the conformational dynamics of the ribosome to transient interactions with those ligands^4^. Until now, however, most studies of translation kinetics have been performed using reconstituted in vitro systems. Although extremely powerful, studies of an isolated system can never fully account for the complexity emerging from inter-connected molecular processes occurring in the crowded environment of the living cell. The development of new methods that allow direct measurements of mRNA translation kinetics inside the cell is, hence, crucial for a complete understanding of mRNA translation and its regulation.

In vivo super-resolution based single-molecule tracking techniques have been successfully used to differentiate functional binding states of key macromolecules (recently reviewed in e.g. ref. ^5^). Initial applications of this approach have mostly been limited to the identification of cellular localizations of molecules in different functional states, whereas the dynamics of the processes has been out of reach due to limited photostability of the fluorescent proteins which have dominated the field of live-cell fluorescence microscopy. The development of new approaches to label biomolecules of interest, exploiting the brightness and photostability of small organic dyes, has opened up new possibilities for live-cell kinetics studies. Such approaches include, e.g., tracking of single dye-labeled tRNAs for translation kinetics measurements^6^, and measurements of spatiotemporal dynamics and translational activity of individual mRNAs^7–12^. The latter was accomplished by introducing arrays of labeling sites on both mRNA and nascent peptides, hence achieving multiple-fluorophore labeling of active translation sites.

In the present work, we introduce a system to directly study in vivo kinetics of translation initiation and elongation. We demonstrate that we can readily detect subtle changes in translation initiation kinetics as a response to antibiotics treatment or due to modifications in the ribosome itself. Our results further shed light on long-standing questions regarding how the bacterial ribosome finds the correct translation start site, and how polycistronic mRNAs are translated in the bacterial cell.

## Results

### Construction of strains for ribosome labeling

**Fig. 1:**
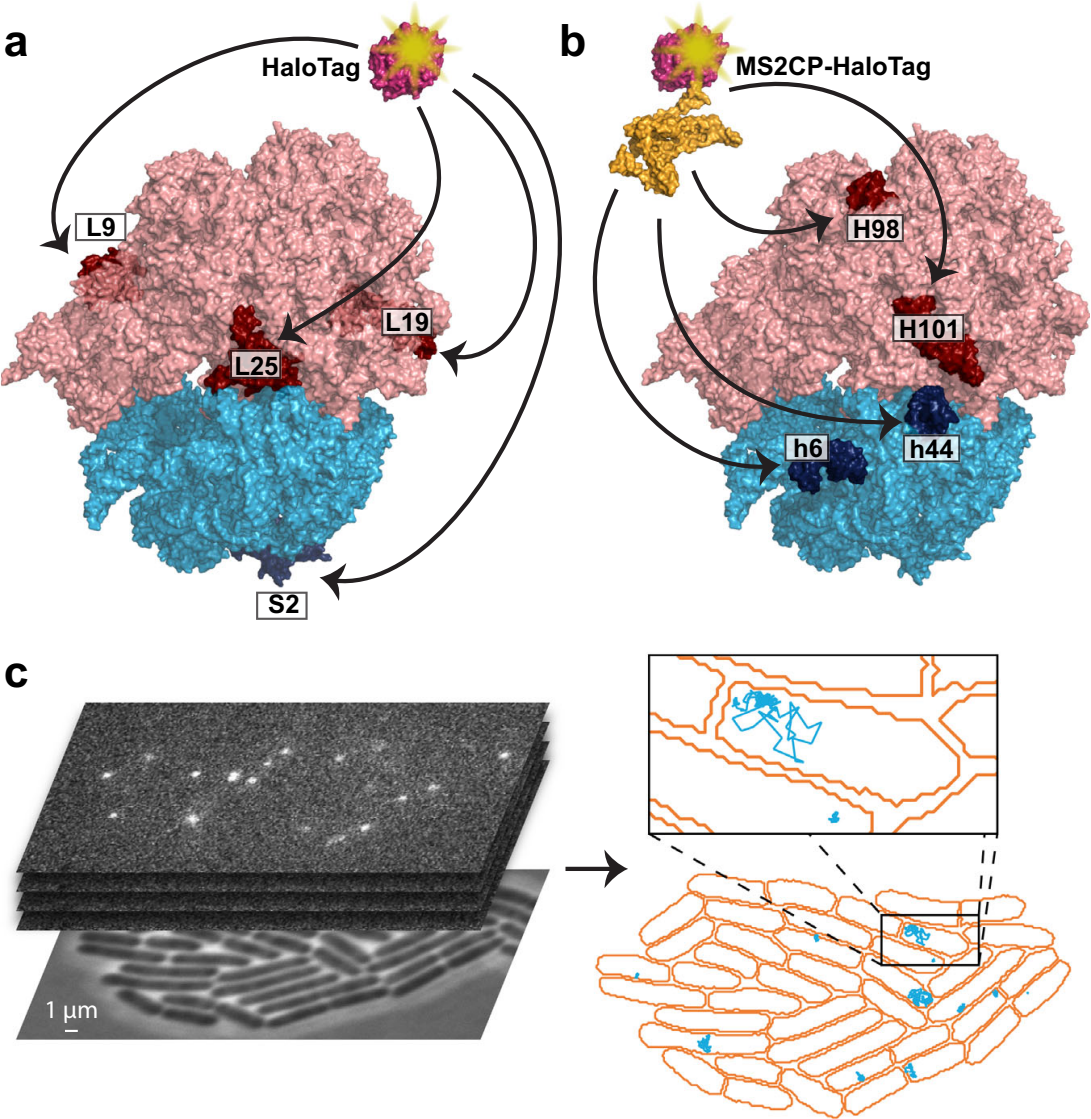
Single-molecule tracking of ribosomal subunits. **a** Site-specific labeling of ribosomal subunits was achieved by genetically fusing the HaloTag protein to a ribosomal protein (L1, L9, L19, or L25 of the 50S subunit, and S2 of the 30S subunit). L1 is not resolved in the shown structure, and hence not highlighted. **b** Site-specific labelling of only a subpopulation of ribosomal subunits was achieved by introducing the MS2 RNA aptamer into a surface exposed helix of ribosomal RNA (23S rRNA H98 or H101, 16S rRNA h6 or h44), to which a co-expressed MS2CP-HaloTag fusion protein binds. **c** Fluorescence time-lapse movies of cells were acquired, and diffusion trajectories of labeled ribosomal subunits were automatically detected in cells segmented based on phase-contrast images.

To track 30S and 50S ribosomal subunits in live *Escherichia coli* we constructed strains in which the gene for HaloTag^13^ was inserted at the C-terminus of the chromosomal locus for ribosomal proteins L1, L9, L19, L25, or S2 (Fig. 1a). The strain expressing L9-HaloTag showed no or minor negative effect on cell growth, cells in which L25 and S2 were mutated showed slower growth, while strains expressing L19-HaloTag or L1-HaloTag showed pronounced growth defects (Supplementary Fig. 1a). In this approach, although all ribosomal subunits are associated with the HaloTag protein, the concentration of the HaloTag ligand can be adjusted to achieve fluorescence labeling of only a small fraction of the ribosomes, simplifying single-molecule tracking. Labeling of ribosomes by means of ribosomal proteins fused to the HaloTag, hence, provides a tool for studying properties of the total pool of ribosomal subunits present in cells. This approach, however, lacks discriminative power when only a subpopulation of ribosomes with specific properties is of interest. In several studies it has been shown that mutated ribosomes with desirable modifications in the rRNA can be isolated by introducing an MS2 RNA aptamer into the rRNA, which is then used for affinity purification by coupling to the strongly interacting MS2 coat protein (MS2CP)^14–16^. Hence, to be able to investigate translation kinetics of only a subpopulation of ribosomes, we inserted the MS2 RNA aptamer in different surface-exposed helices of the 16S rRNA or 23S rRNA (Fig. 1b) of an *rrnB* operon copy located on a plasmid. To test whether the insertion interferes with the production or activity of ribosomes, we introduced the different plasmids with modified *rrnB* operons into the *E. coli* SQ171 strain, which lacks all chromosomal rRNA operons. In agreement with previous studies, we found that extension of h6 of the 16S rRNA and H98 of 23S rRNA did not affect the growth rate of the cells^15^, whereas cells with modified 16S rRNA h44 and 23S rRNA H101 showed significant growth defects (Supplementary Fig. 1b). In previous studies, extensions of h44 and H101 have been used for in vitro fluorescence labelling of ribosomal subunits^17^. Although additive negative effects of these extensions on the cell growth rate was found, it was concluded that the function of the modified subunits was not significantly affected. We note that the observed growth defect might actually be caused by interference during ribosome biogenesis, rather than perturbed function of the mature ribosomes. However, for our studies we used only h6- and H98-modified ribosomes.

We next assessed the effect of the MS2CP-HaloTag expression on cell growth in the *E. coli* SQ171 strain producing either unmodified ribosomes or h6- and H98-modified ribosomes (Supplementary Fig. 2). Expression of MS2CP-HaloTag resulted in dosage-dependent toxicity on the cells expressing the wt *rrnB* operon, likely caused by unspecific binding of MS2CP-HaloTag to cellular RNAs^18^. However, no or only a slight growth defect was observed upon MS2CP-HaloTag expression in cells containing h6-modified ribosomes with an MS2CP binding site. This result shows that the otherwise toxic MS2CP-HaloTag is efficiently sequestered by the modified ribosomes, and further, that such ribosomes are fully functional when bound to MS2CP-HaloTag. In the cells producing H98-modified ribosomes, expression of MS2CP-HaloTag showed a small but noticeable dosage-dependent negative effect on the cell doubling time. This indicates that binding of MS2CP-HaloTag to such ribosomes has a slight negative impact on their function.

### Tracking of single fluorescent 30S and 50S subunits in live cells

For single-molecule tracking experiments we selected the L9-HaloTag and S2-HaloTag strains, as well as strains containing the plasmid for expression of h6-modified 16S rRNA or H98-modified 23S rRNA and a plasmid encoding the MS2CP-HaloTag fusion. From our cell growth assays (Supplementary Fig. 1 and 2), however, we note that the L9 labeled 50S strain, and the h6 labeled 30S strain, most likely represent the wild-type situation best. To fluorescence label the molecules fused to the HaloTag, cells in exponential growth phase were incubated with the JF549 HaloTag ligand which penetrates into the *E. coli* cells and rapidly and specifically forms a covalent bond with the HaloTag protein^19^. The concentration of the JF549 HaloTag ligand present during labeling was optimized to achieve conditions where only a few fluorescently labeled molecules were present in each cell. After extensive washing, the cells were sparsely spread on an agarose pad, grown to mini colonies, and imaged at 37 °C (Fig. 1c). For all strains tested, we observed that practically all (~99%) cells continued growth on the agarose pad and contained fluorescently labeled molecules. Labeled molecules were stable for several hours and were homogeneously distributed within mini colonies produced from each single labeled cell as a result of cell division (Supplementary Fig. 3). Mini colonies were imaged using stroboscopic laser illumination^20^ with a 3 ms laser pulse per 30 ms camera exposure frame. The resulting microscopy data obtained from more than 100 individual colonies were processed using our previously described pipeline for automatic cell segmentation, fluorescent molecules detection, and building of single-molecule diffusion trajectories^6^ (Fig. 1c). The apparent average diffusion coefficient for fluorescently labelled particles was very similar in all the selected strains (Supplementary Fig. 4a) and consistent with the expected diffusion for ribosomal particles^21,22^, i.e., approximately 0.1 µm^2^/s. As a control, we also tracked free labeled HaloTag proteins in the genetic background strain, as well as MS2CP-HaloTag in cells expressing the *rrnB* operon without insertion of the MS2 aptamer. In both these cases, the apparent average diffusion coefficient of the labeled molecules was approximately two orders of magnitude higher (Supplementary Fig. 4b), matching what was previously reported for freely diffusing fluorescent proteins^23^. Hence, the HaloTag fusion to ribosomal proteins allows specific labeling of ribosomal subunits, whereas the presence of the MS2 aptamer in 16S rRNA or 23S rRNA allows specific labeling of a subpopulation of 30S or 50S for in vivo tracking.

### HMM analysis of diffusion trajectories

The high brightness and stability of the JF549 dye allowed us to track ribosomal subunits for up to hundreds of frames, with an average of around 40 frames per trajectory (Supplementary Fig. 5), which lead to the direct observation that the ribosomal particles do not diffuse homogeneously over time, but rather persist in different long-lived relatively discrete diffusional states (Supplementary Movie 1).

To obtain quantitative information on diffusion states and the transitions between these, we analyzed the diffusion trajectories using a Hidden Markov Modeling (HMM) approach^24^ previously used to extract ribosome binding kinetics of fluorescently labelled tRNAs^6^. Here, all trajectories are fitted to a pre-set number of diffusion states, using the respective diffusion coefficients (*D*) and the transition frequencies between these states as fitting parameters. The algorithm searches for global maximum likelihood estimates (MLE) of the fitted parameters, and accounts for motion blur and localization uncertainties. Based on the results from previous single-molecule tracking of ribosomal subunits labeled with fluorescent proteins^21,22^, we expected that the HMM analysis should be able to distinguish freely diffusing ribosomal subunits from translating subunits bound to mRNA. However, we also anticipated that the ‘bound’ state, in particular, might not be specified by a single diffusion rate, but rather display a continuum of diffusion states representing everything from a comparably rapidly diffusing single 30S or 70S bound to an mRNA, to slowly diffusing polysomes, perhaps also tethered to the chromosome or bound to translocons in the cell membrane^21,25–27^. Hence, the diffusion trajectories were fitted to models with 2–11 discrete diffusion states. The average diffusion coefficient distributions (calculated separately based on mean-squared displacement (MSD)) are very similar between independent experiments (Supplementary Fig. 6), and we observe good overall reproducibility in the diffusion states assignments by HMM (Supplementary Data 1–3). To achieve convergence in the kinetic rate estimates, however, all results presented below are calculated from cumulated data from several independent experiments (2–7).

**Fig. 2:**
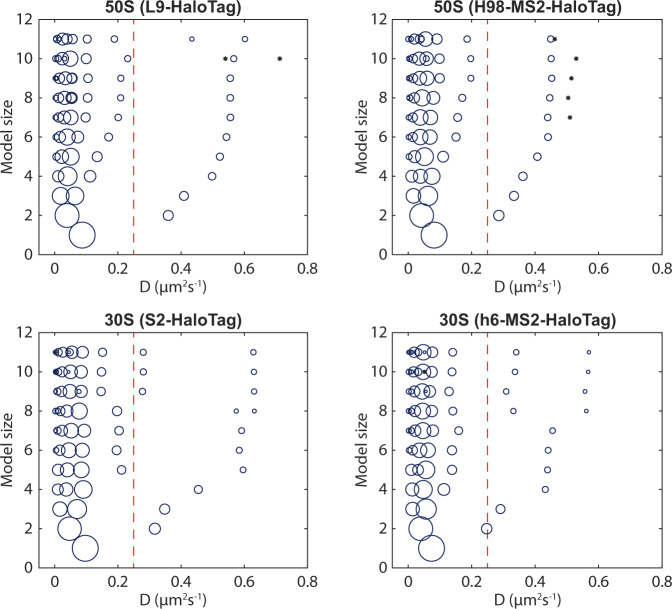
HMM-estimated occupancies in different diffusion states for all fitted model sizes. The area of the circles is proportional to the relative occupancy. Occupancies lower than 1% are shown as *. The red dashed lines indicate the threshold, 0.25 µm^2^/s, used for coarse-graining larger models to a two-state model. All datasets are also shown in Supplementary Data 4–7. *n* = 179569, 115349, 161623, and 191950 trajectory steps collected from 3, 3, 7, and 6 independent experiments for L9, H98, S2, and h6 labelling, respectively. Source data are provided as a Source Data file.

For both the 30S and 50S tracking data, independent of model size, we find the largest fraction of ribosomal subunits diffusing at 0.03–0.06 µm^2^/s, and a smaller fraction at around 0.3–0.7 µm^2^/s (Fig. 2, Supplementary Data 4–7). In models with more HMM states, the slower state is divided into several states, in agreement with our expectation of a loosely defined ‘bound’ state.

It should be noted that the used HMM algorithm does not provide any information about which model size is most parsimonious, as does the commonly used vbSPT algorithm^28^. However, as vbSPT tends to overfit data^29,30^, and more importantly, does not account for motion blur and localization uncertainties, we prefer to rely on the current algorithm, and instead reduce the model sizes by coarse-graining into biologically relevant diffusion states, as discussed below. Allowing a large number of diffusive states, which are then coarse-grained, also solves potential problems with non-exponential state-time distributions^27^. That is, since the HMM fitting procedure assumes exponentially distributed transition times between states, which is not expected for productive bindings of ribosomal subunits to mRNAs, the fitting of several inter-connected states, with the same or similar diffusion rate constant, will allow the overall dwell time, e.g., in the mRNA bound state, to have non-exponential distribution. We also note that bigger model sizes fit the data better according to Akaike’s information criterion (Supplementary Fig. 7).

### Diffusion of free ribosomal subunits

Efficient initiation of translation for most *E. coli* genes depends on the presence of the Shine-Dalgarno (SD) motif, with a consensus sequence GGAGG located upstream of the AUG start codon. The SD motif facilitates recruitment of the 30S subunit via RNA-RNA base-pairing with the corresponding CCUCC motif at the 3’ terminus of the 16S rRNA, referred to as the anti-Shine-Dalgarno sequence (ASD)^31^. Alterations in the ASD have been shown to render 30S subunits unable to efficiently initiate translation of endogenous mRNAs, while mRNAs with the corresponding modifications in the SD motif were specifically translated only by such mutated, orthogonal, ribosomal subunits^32,33^. Hence, to specifically study the diffusion of unbound 30S subunits, we introduced the MS2 RNA aptamer into h6 of the orthogonal 30S (O-30S)^33^, and co-expressed these with the MS2CP-HaloTag fusion in a strain background without any corresponding orthogonal mRNA.

We noticed that expression of O-30S subunits was moderately toxic for the *E. coli* cells, and that a C1400U mutation in the 16S rRNA of the O-30S appeared frequently during cultivation, which alleviated this toxicity. The C1400 residue in 16S rRNA is situated at the center of the decoding site of 16S rRNA, and other mutations of C1400 in ASD-altered ribosomes greatly impact negatively the overall activity of the ribosomes for both endogenous mRNAs and orthogonal mRNAs^34^. Hence, it is likely that O-30S subunits are still significantly involved in translation of endogenous genes, perturbing the cell proteome, and that the C1400U mutation reduces this activity^34–36^.

**Fig. 3:**
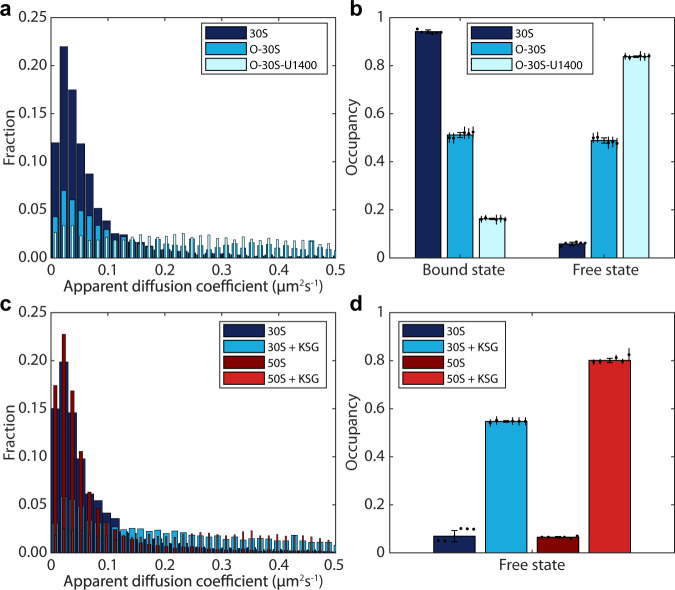
Distinction between freely diffusing and mRNA-bound ribosomal subunits. **a** Distribution of apparent diffusion coefficient for 30S variants (h6 labelling), estimated from mean-squared-displacement analysis of diffusion trajectory segments of 7 frames. **b** HMM-estimated occupancy (steady-state fraction) of 30S subunit variants (h6 labelling) in mRNA-bound or freely diffusing state. *n* = 191950, 31023, and 43010 trajectory steps collected from 6, 2, and 3 independent experiments for 30S, O-30S, and O-30S-U1400, respectively. **c** Distribution of apparent diffusion coefficient for ribosomal subunits (S2 and L9 labelling) in cells untreated, or treated with 2 mg/ml KSG. **d** HMM-estimated occupancy of 30S and 50S subunits (S2 and L9 labelling, respectively) in the freely diffusing state in presence or absence of 2 mg/ml KSG. *n* = 161623, 43846, 179569, and 63265 trajectory steps collected from 7, 2, 3, and 2 independent experiments for 30S, 30S+KSG, 50S, and 50S+KSG, respectively. Panels b and d show weighted averages of coarse-grained HMM results from 7–11 state models, using 0.25 µm^2^/s as cutoff between bound and free subunits (Supplementary Data 12). The complete results for all model sizes (2–11) are shown in Supplementary Data 4 and 6–11. Error bars with caps represent weighted standard deviation, calculated from individual model sizes (7–11) with bootstrap estimated standard errors (shown as error bars without caps). Source data are provided as a Source Data file.

We tracked both O-30S and O-30S-U1400 subunits and compared their diffusion with that of normal 30S subunits. The average diffusion of O-30S and O-30S-U1400 was found to be 0.37 µm^2^/s and 0.46 µm^2^/s, respectively (Fig. 3a). This is significantly higher than 0.073 µm^2^/s as observed for 30S subunits with unaltered ASD (Fig. 3a), suggesting that, indeed, both types of O-30S subunits bind mRNA to a lesser extent. From the HMM analysis of O-30S trajectories we find that, for all model sizes, the occupancy (steady-state fraction) in the faster diffusion states increases dramatically compared to normal 30S subunits. By comparing the HMM results for O-30S-U1400, with those of the 30S with unaltered ASD, we find that a threshold at around 0.15–0.3 µm^2^/s would enable us to separate freely diffusing ribosomal subunits from 30S participating in translation (Supplementary Fig. 8). With the threshold at 0.25 µm^2^/s we observe that about 50% and 85% for O-30S and O-30S-U1400, respectively, are in the fast diffusion state, compared to less than 10% for ribosomes with unaltered ASD (Fig. 3b, Supplementary Data 7–9 and Supplementary Fig. 11). The difference in diffusion for O-30S and O-30S-U1400, further suggests that O-30S subunits partially retain their ability to bind and translate endogenous mRNAs, in agreement with results of ribosomal profiling for orthogonal ribosomes^36^, and that mutation of U1400 dramatically reduces their affinity to mRNA.

To further validate that the threshold at 0.25 µm^2^/s can be used to distinguish free and bound subunits, and to investigate whether the same threshold can be used for 50S subunits, we tracked 30S and 50S in cells treated with a high concentration of kasugamycin (KSG), an aminoglycoside antibiotic that specifically targets translation initiation^37^.

*E. coli* cells containing either L9-HaloTag or S2-HaloTag were grown on an agarose pad for 2 h, after which the pads were soaked in media containing 2 mg/ml KSG. From the HMM analysis of diffusion trajectories in cells subjected to KSG treatment, we observe that the diffusion rate distribution for both 30S and 50S particles changed radically compared to untreated cells, with average diffusion coefficients at 0.34 µm^2^/s and 0.46 µm^2^/s (Fig. 3c), respectively. Comparison of HMM results for 50S subunit in cells treated with KSG and untreated cells suggests that free 50S subunits diffuse with *D* > 0.35 µm^2^/s and that a threshold at around 0.25–0.35 µm^2^/s would enable us to separate freely diffusing 50S from 50S participating in translation (Supplementary Fig. 9). For 30S we obtained results that are similar to those of ASD-modified ribosomes, with a large increase in occupancies in diffusional states >0.35 µm^2^/s upon KSG treatment (Supplementary Fig. 9). With the threshold at 0.25 µm^2^/s we observe that ~80% and ~55% for 50S and 30S, respectively, are in the fast diffusional states in cells treated with KSG (Fig. 3d and Supplementary Data 10–12). It has been shown in vitro that KSG only moderately affects the binding of the 30S subunit to an mRNA, but strongly reduces its affinity to initiator fMet-tRNA^fMet^ ^38^. Therefore, it is expected that the 30S subunit should be able to bind mRNA even in the presence of KSG and will, hence, show overall slower diffusion. However, such complexes are incompetent in binding 50S, due to the lack of fMet-tRNA^fMet^, which is likely the reason why we observe a larger proportion of 50S than 30S in the free diffusion state upon KSG treatment.

We conclude that a threshold at 0.25 µm^2^/s enables us to separate both 30S and 50S free subunits from those bound to mRNA.

### Kinetics of translation elongation and initiation

**Fig. 4:**
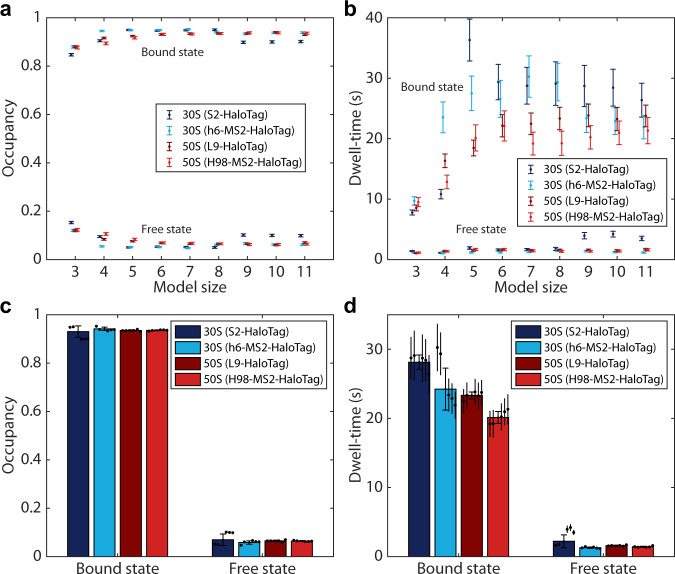
Translation kinetics from coarse-grained HMM-results. **a, b** Estimated occupancy and dwell-time of ribosomal subunits in the mRNA-bound state or the freely diffusive state for different HMM fitting model sizes. Full models (Supplementary Data 4−7) were coarse-grained into two states: mRNA-bound or free, using the threshold 0.25 µm^2^/s. **c, d** Weighted averages of occupancy and dwell-time, based on model sizes 7–11 (as shown in panels **a**, **b**, Supplementary Data 12). Error bars with caps represent weighted standard deviation, calculated from individual model sizes (7–11) with bootstrap estimated standard errors (shown as error bars without caps). *n* = 179569, 115349, 161623, and 191950 trajectory steps collected from 3, 3, 7, and 6 independent experiments for L9, H98, S2, and h6 labelling, respectively. Source data are provided as a Source Data file.

In addition to the occupancy in each diffusion state, the HMM analysis also provides the estimated transition frequencies between these states, or conversely, the average dwell-time in each state. To study specifically the transitions between free subunits and mRNA-bound subunits, we coarse-grained the HMM models of each size obtained for 50S subunits (L9-HaloTag and H98-MS2-HaloTag) and 30S subunits (S2-HaloTag and h6-MS2-HaloTag) into two states in accordance with the control experiments presented in the previous section: mRNA bound ribosomal subunits at *D* < 0.25 µm^2^/s and freely diffusing subunits with *D* > 0.25 µm^2^/s. Both the occupancy and the dwell-time in the coarse-grained functional states converge well for both 30S and 50S subunits with increasing model size (Fig. 4a–b). By varying the threshold, we find the four datasets to be sensitive to different extent, with the 50S results in general less threshold dependent than the 30S results, and the bound-state parameters more robust than the free state parameters (Supplementary Fig. 10 and Supplementary Data 13). We also find that a threshold at 0.25 µm^2^/s minimizes the bootstrap estimated errors for dwell-times (Supplementary Fig. 11). For simplicity and clarity, in the following discussions we present weighted average values of occupancies and dwell-times, calculated from coarse-grained data (using a threshold of 0.25 µm^2^/s) of model sizes 7–11 (Supplementary Data 12). Whereas absolute numbers are slightly different between different model sizes, our conclusions do not depend on exact model size ≥ 7 states. HMM estimated parameters are further the same (within 8%) for thresholds 0.25–0.40 µm^2^/s, for 50S subunits, and 0.20–0.30 µm^2^/s, for h6-labelled 30S subunits (Supplementary Fig. 10). For the S2 labelled 30S, we find that a threshold change of 0.05 µm^2^/s, up or down, results in significant changes in the free-state parameters (up to 80%), whereas the bound-state parameters remain within 12% variation.

To investigate the kinetics of translation inside rapidly growing *E. coli* cells, we first compare the steady-state fraction of 30S and 50S subunits in the free or mRNA-bound diffusion states. For both 30S and 50S subunits, with both labeling strategies, we find that 93–94% are at any given moment engaged in translation (Fig. 4c). Hence, the fraction of free subunits, 6–7%, is lower than what has previously been reported using other methods (e.g., 15%^39^). However, it has also been estimated that precursors of 30S and 50S subunits comprise approximately 10% of all subunit particles^40^. Since in our experiments, data were acquired more than 2 h after labeling, we most probably track only the diffusion of fully assembled and processed particles. Hence, from our analysis we estimate that over 90% of the mature fraction of both subunits are actively translating at any given moment.

From the HMM estimated transition frequencies of the ribosomal subunits between the diffusion states, we find that the 50S subunits spend, on average, 20 s+/−1 s, or 23 s+/−1 s bound to an mRNA, and the time spent freely diffusing between binding events is on average 1.4 s+/−0.1 s or 1.6 s+/−0.1 s depending on the labeling strategy (Fig. 4d). For the h6-labeled 30S subunit, we observe very similar estimates to those of 50S (24 s+/−3 s and 1.3 s+/−0.1 s), whereas for S2 labeled 30S the corresponding numbers are slightly higher (28 s+/−1 s and 2.2 s+/−1 s) (Fig. 4d). The S2 protein is involved in binding of S1 ribosomal protein which is essential for translation initiation from most of the mRNAs. The growth of the S2-labeled strain was also most prominently affected among the four main strains used in the present study (Supplementary Fig. 1) and we observed an altered phenotype for it (Supplementary Fig. 12). Hence, it is possible that the kinetics of translation is affected in this strain.

The fact that the mRNA-bound state dwell-time is similar for both subunits (Fig. 4d), has interesting consequences. More than 50% of all *E. coli* genes are organized in operons which are transcribed into polycistronic mRNA transcripts^41,42^. Polycistronic organization of the transcriptome has been shown to be important for translational coupling of genes for which exact stoichiometry is required for correct operation^43–45^. Such coupling has been suggested to occur through either or both of two hypothetical mechanisms^46^. In the first case, translation of a downstream gene by a ribosome only occurs when another ribosome is translating the upstream gene, thereby unwinding a stable mRNA structure, which would otherwise block the ribosome binding site of the downstream gene^46^. In the second scenario, initiation on the downstream gene occurs by the very same 30S^47^ or 70S^48^ which translated the upstream gene, without dissociation from the mRNA. Our estimates of similar dwell-time in the mRNA bound diffusion state for both the small and the large ribosomal subunits (Fig. 4d), strongly suggest that 30S re-initiation is not prevalent for translation initiation optimization.

Considering that elongating ribosomes have been estimated to proceed at a speed of on average 16-17 amino acids per second under the growth conditions of the present experiments, i.e., 2 doublings/h^49,50^, a ribosome would need 14−15 s to translate a ‘typical’ *E. coli* protein with a length of around 240 amino acids (estimation based on proteomics data^51^, see Supplementary Data 14). Our estimate for the dwell-time in the slow diffusion state is 50% longer than this, 23 s and 24 s, for 50S and 30S respectively (L9 and h6 labelling, which have minimal effect on cell growth (Supplementary Fig. 1 and 2)). Hence, our results suggest that 70S ribosomes do re-initiate on downstream ORFs after 50% of the termination events (assuming on average two open reading frames per mRNA^42^). Independent initiation from each open reading frame would dominate the global landscape of translation if i) the actual average elongation rate is significantly lower than 16-17 aa/s and/or ii) the length of the average protein is significantly higher than 240 aa. The experimentally measured average elongation rate was not estimated globally, but rather for selected proteins using the β-galactosidase assay and pulse-chase assays^50^. Hence, the provided estimates, might correspond to the maximal elongation rate (i.e., the first translation events being completed) at certain conditions rather than an average. However, indirect calculations based on the amount of total protein per cell, the number of ribosomes per cell (both of which can be estimated accurately), the percentage of active ribosomes (set at 85%), and the cell growth rate, suggest that the global elongation rate is 21−22 aa/s^52^. This is even higher than what was experimentally measured for selected proteins. Taking into account that the percentage of active elongating ribosomes can only be 10–15% higher, these data strongly suggest that the average elongation rate is unlikely to be lower than 16−17 aa/s at these growth conditions. With regards to the estimates for the length of a “typical protein”, besides proteomic studies, ribosome-profiling has been used to calculate absolute translation frequencies for every known gene in *E. coli*^53^. Analysis of available data from ribosomal profiling is in good agreement with proteomic studies, with length estimates for an average translated protein of around 192–222 aa (Supplementary Data 15). Hence, analyses of available data strongly suggest that a translation time of 14−15 s per open reading frame likely represents an upper bound, supporting that our results of 23−24 s per ribosome-mRNA binding event suggest widespread 70S re-initiation.

It can further be noted that the similar bound-state dwell-times of 30S and 50S suggest that 30S does not, in general, await 50S joining on the mRNA for long periods of time. Hence, if 30S binding to ribosome stand-by sites^54^ is prevalent in *E. coli*, these states are transient, at least in the time scale of the precision of our experiments, 1−2 s (Fig. 4).

### Inhibition of initiation by AtaT toxin and KSG

Our results suggest that the 70S ribosome can continuously translate several open reading frames and re-initiate on downstream ribosome binding sites without dissociating from an mRNA. Hence, when translation initiation is inhibited we would expect that not only the dwell-time in the “free” but also the “bound” diffusion state, of both ribosomal subunits, should increase due to hampered re-initiation. To test this hypothesis, we analyzed the diffusion of 30S and 50S subunits in cells exposed to AtaT. AtaT is a toxin from the recently characterized toxin-antitoxin complex AtaRT, which specifically modifies Met-tRNA^fMet^ by acetylating the free amino group of the methionine^55^. This modification prevents the interaction between tRNA^fMet^ and IF2, which in turn leads to inefficient formation of the 70S initiation complex^55^. Effectively, expression of the AtaT toxin should decrease the concentration of available initiator fMet-tRNA^fMet^ in the cells, which is required for all known types of translation initiation. Hence, strains encoding S2-HaloTag or L9-HaloTag were transformed with a plasmid for low level expression of AtaT, which, during the experiment was induced after 2 h of incubation on the agarose pad.

In addition, we performed experiments with subinhibitory concentrations of KSG. Although KSG interferes with initiator tRNA^fMet^ binding to 30S as well, it is known to inhibit only canonical translation initiation on transcripts with SD motifs, and does not interfere with translation initiation on leaderless mRNAs which are initiated by 70S directly^56^. Moreover, a global analysis of the effect of KSG on the whole translatome showed that downstream genes of polycistronic operons display pronounced resistance to KSG inhibition, suggesting that KSG only inhibits canonical de-novo initiation and not re-initiation^57^. Hence, even if 70S re-initiation is common in the cell, KSG should not extend the bound-state dwell-time of 50S, as AtaT is expected to do. To perform this experiment, strains encoding S2-HaloTag and L9-HaloTag were grown on agarose pads for 2 h, after which the pads were soaked in media containing 20 µg/ml KSG (~0.15 times MIC).

**Fig. 5:**
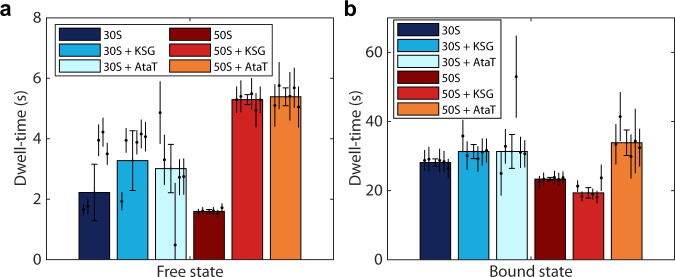
Inhibition of initiation by AtaT toxin and KSG. **a**, **b** HMM-estimated dwell-time of 30S and 50S subunits in the freely diffusing state (**a**) or in the mRNA bound state (**b**) in presence or absence of 20 µg/ml KSG or during the expression of the toxin AtaT. The data shows weighted averages from coarse-grained HMM-fitted model sizes 7–11 (Supplementary Data 12). Results for individual model sizes (2–11) are shown in Supplementary Data 4, 17−21. Error bars with caps represent weighted standard deviation, calculated from individual model sizes (7–11) with bootstrap estimated standard errors (shown as error bars without caps). *n* = 161623, 141242, 101518, 179569, 182163, and 103391 trajectory steps collected from 7, 2, 5, 3, 2, and 5 independent experiments for 30S, 30S+KSG, 30S+AtaT, 50S, 50S+KSG, and 50S+AtaT, respectively. Source data are provided as a Source Data file.

From the HMM analysis of diffusion trajectories of 50S in cells either producing AtaT toxin or subjected to KSG treatment, we observe that the dwell-time in the free diffusion state increases significantly, from 1.6 s+/−0.1 s in untreated cells to 5.4 s+/−0.3 s and 5.3 s+/−0.2 s, respectively (Fig. 5a, Supplementary Data 12, 16, 17). These results show that in both cases, 50S association to the mRNA bound 30S subunit is delayed, and we achieve similar levels of de-novo initiation inhibition. We also find that the occupancies in the free state for 50S increase upon treatment with AtaT and KSG, from 6.5%+/−0.3% to 15.2%+/−0.8% and 23.7%+/−1.0%, respectively (Supplementary Fig. 13, Supplementary Data 12, 16, 17). The dwell-time in the free state for 30S increased to a lesser extent, if at all, from 2.2 s+/−0.9 s in untreated cells to 3.0 s+/−0.8 s and 3.3 s+/−1.0 s, in cells subjected to AtaT and KSG, respectively (Fig. 5a, Supplementary Data 12, 18, 19). This is expected, since 30S can bind mRNA in the absence of other factors, and KSG has been shown to affect binding of 30S to mRNA only moderately^38^. Similarly, we also find that the occupancies in the free state for 30S increase moderately in cells treated with AtaT and KSG, from 7.0%+/−2.4% to 11.1%+/−0.8% and 10.6%+/−3.0%, respectively (Supplementary Fig. 13, Supplementary Data 12, 18, 19). Strikingly, we observed a significant increase for the 50S dwell-time in the mRNA bound state for AtaT producing cells, from 23.3 s+/−0.1 s to 33.9 s+/−3.7 s, but not in KSG treated cells (19.3 s+/−1.5 s) (Fig. 5b, Supplementary Data 12, 16, 17). Since only AtaT is expected to inhibit the presumptive re-initiation on poly-cistronic mRNAs, these results speak in favor of the 70S re-initiation model. We also observed a noticeable increase for the dwell-times of 30S in the bound state, from 28.1 s+/−1.0 s in untreated cells to 31.3 s+/−2.0 s in cells subjected to KSG, showing that the 30S subunit remains bound to an mRNA awaiting 50S association (Fig. 5b, Supplementary Data 12, 19). Although we also observed increased mean dwell-time for the 30S bound state for cells producing AtaT (Fig. 5b, Supplementary Data 12, 18), the difference is not as big as for 50S, which would be expected. We note, however, that the HMM results are more model size-dependent for all three 30S datasets presented compared to the 50S datasets (Fig. 5b, Supplementary Data 12, 18), and we recall that the HMM analysis of the 30S trajectories are more sensitive to the coarse-graining threshold (Supplementary Fig. 10).

To conclude, our results with the AtaT toxin are in perfect agreement with our hypothesis that inhibition of any type of initiation should increase the bound-state dwell-time of 50S, which provides additional support for the 70S re-initiation model. Furthermore, our data support the growing evidence that KSG inhibits only canonical initiation and does not inhibit initiation by 70S ribosomes, which occurs on leaderless mRNAs and appears to be the major mechanism for translation re-initiation on poly-cistronic mRNAs.

### Temperature-dependent translation elongation rates

**Fig. 6:**
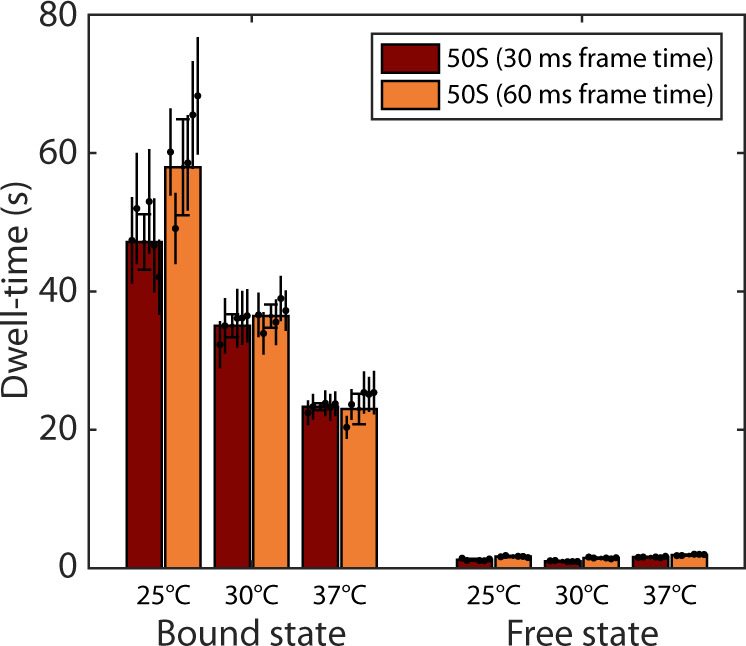
Estimated mRNA-bound-state dwell-time of 50S subunits at different temperatures and with microscopy data acquired at 30 or 60 ms camera exposure times. The data shows weighted averages from coarse-grained HMM-fitted model sizes 7–11 (Supplementary Data 12). Results for individual model sizes (2–11), are shown in Supplementary Data 4 and 20−24. Error bars with caps represent weighted standard deviation, calculated from individual model sizes (7–11) with bootstrap estimated standard errors (shown as error bars without caps). *n* = 142400, 146595, 144083, 155262, 179569, and 93809 trajectory steps collected from 2, 2, 2, 3, 3, and 2 independent experiments for 30 and 60 ms frame time at 25 °C, 30 °C, and 37 °C respectively. Source data are provided as a Source Data file.

In the interval between 25 °C and 37 °C, the translation elongation rate increases proportionally to the cell growth rate^58^. Previous studies have suggested that the elongation rate at 25 °C and 30 °C is decreased to ~35% and ~70%, respectively, of the elongation rate at 37 °C. To further validate that our method can be used for measurements of translation elongation and initiation rates, we tracked L9-labeled 50S subunits at these temperatures. With increasing HMM model sizes, the dwell-times in the bound state for experiments performed at 25 °C and 30 °C converge to 47 s+/−4 s and 35 s+/−2 s, respectively (Fig. 6, Supplementary Data 12, 20, 21). While the measured relative change in elongation rate at 30 °C is well in agreement with previous studies, we likely obtain underestimated values for the average elongation time measured at 25 °C due to very rare transitions between molecules in bound and free states. We therefore performed additional experiments in which we tracked L9-labeled 50S subunits at different temperatures using a two times lower data acquisition rate with which we should capture more transitions in a dataset of the same size. We find that the estimated dwell-times in the bound state are indistinguishable between experiments performed at different frame rates at 30 °C and 37 °C (Fig. 6, Supplementary Data 4, 21, 23, 24, and summarized in Supplementary Data 12). However, in experiments with lower frame rate at 25 °C we find the average bound time to be 58 s+/−7 s, which is in better agreement with previous studies (Supplementary Data 12, 20, 22). Hence, we conclude that our ribosome tracking approach can accurately estimate translation elongation rates in a wide range. Interestingly, the time the 50S subunit spends freely diffusing between binding events is similar or shorter at 25 °C (1.2 s+/−0.1 s) and 30 °C (1.0 s+/−0.1 s) than at 37 °C (1.6 s+/−0.1 s). In comparison to the elongation stage, which is partly rate limited by the highly temperature-dependent step of peptidyl transfer^59^, initiation mainly depends on association of macromolecules, which in turn is limited by the less temperature-sensitive diffusion. Possible explanations for slightly faster subunit joining could be an increase of available initiation factors due to a lower fraction of free ribosome subunits, and/or that the 30S subunit finds the correct start site faster due to stronger SD-ASD interaction at lower temperatures.

### Selection of initiation sites by 30S subunits

In *E. coli*, initiation sites on mRNAs can be recognized by 30S alone^60^. In certain cases, diffusion of 30S along an mRNA has been shown to promote translation initiation^47^, but it is generally assumed that the 30S subunit binds directly or in very close proximity to the initiation site. Base pairing between the SD and the ASD should, in principle, direct the 30S to the start codon. However, a recent study employing ribosomes with a modified ASD sequence, suggested that the SD-ASD interaction is not enough for efficient translation, and that initiation sites are rather hardwired by other mRNAs features^36^. It has also been shown that the initial association of 30S with mRNAs depends on secondary structures nearby the initiation site, and is independent of the SD motif and start codon, which are monitored in subsequent steps^61,62^. Hence, it is still debated to what extent initiation relies on the SD-ASD interaction in *E. coli* and how widespread this mechanism of modulation of translation initiation is in other bacteria^63,64^.

In the experiments described above, which helped us to establish the threshold for separation of free and mRNA-bound 30S subunits, we noticed that the previously developed O-30S^33^ appear to be significantly involved in binding of endogenous mRNAs (Fig. 3a, b). Besides a mutated ASD sequence, these ribosomes carry the additional 16S rRNA substitutions G722C and U723A located in the vicinity of the helix formed by SD and ASD, as well as a C1192U mutation which confers spectinomycin resistance^65^. To investigate the contribution from the ASD sequence alone, as well as from these additional rRNA mutations, we analyzed the occupancy and dwell-times of several modified 30S subunits in the freely diffusing state and the mRNA-bound state using the coarse-grained HMM-analysis as described above (Supplementary Data 25−28 and summarized in Supplementary Data 12). To minimize the effect of toxicity associated with expression of ASD-modified ribosomes, these experiments were performed with only low-level expression of the mutated rRNA operons.

**Fig. 7:**
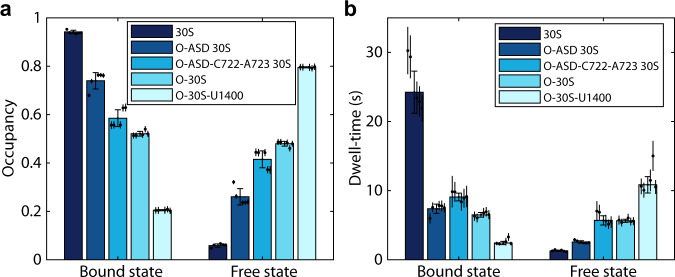
Translation kinetics of modified 30S subunits. Estimated occupancy (**a**) and dwell-time (**b**) of tracked 30S subunit variants in the mRNA-bound state or the freely diffusive state. The data shows weighted averages from coarse-grained HMM-fitted model sizes 7–11 (Supplementary Data 12). Results for individual model sizes (2–11) are shown in Supplementary Data 7 and Supplementary Data 25−28. Error bars with caps represent weighted standard deviation, calculated from individual model sizes (7–11) with bootstrap estimated standard errors (shown as error bars without caps). *n* = 191950, 90335, 33838, 86221, and 55186 trajectory steps collected from 6, 6, 2, 2, and 2 independent experiments for 30S, O-ASD 30S, O-ASD-C722-A723 30S, O-30S, and O-30S-U1400, respectively. Source data are provided as a Source Data file.

For 30S subunits in which only the ASD sequence was mutated (O-ASD), a substantial fraction (74%) was still found to be in the mRNA-bound state at any given moment, although corresponding O-SD mRNAs were not present in the cells. Incorporation of the G722C-U723A mutations (O-ASD-C722-A723) and the additional C1192U mutation (O-30S) decreased this fraction further, to 59% and 52% respectively, whereas the additional C1400U mutation (O-30S-U1400) finally brought the bound-state occupancy down to a mere 21% (Fig. 7a).

From the HMM estimated transition frequencies between the free and mRNA-bound diffusion states, we find that O-ASD, O-ASD-C722-A723, and O-30S spend, on average, approximately the same 7–9 s in the mRNA-bound state, whereas the time spent freely diffusing between binding events is between 3 s and 6 s (Fig. 7b). The bound state for these mutants, although relatively long-lived, is shorter than what we have measured for unmodified 30S subunits (24 s, Figs. 4d and 7b). Early works and recent ribosome profiling studies show that ASD-modified ribosomes in the absence of orthogonal mRNA (i) are found in a polysomal fraction, (ii) form 70S complexes on mRNA, and (iii) produce long polypeptide chains, altogether, supporting that they do translate endogenous transcripts to a significant extent^34,36^. Furthermore, analysis of ribosome footprint distributions in the absence and presence of retapamulin, an antibiotic that specifically traps 70S initiation complexes on initiation sites, strongly suggest that such ribosomes initiate from the same ribosome binding sites as wild-type ribosomes, although with different translation efficiencies^36^. It is, however, unlikely that much shorter bound dwell times for ASD-modified ribosomes, can be explained solely by changes in translation efficiencies of different genes. We propose that several factors contribute to the shorter bound-state dwell-times we find for orthogonal 30S variants. First, despite the fact that ASD-modified ribosomes are able to locate ribosome binding sites, initiation may occur from an incorrect start codon with a shifted reading frame which can result in translation of nonsense short polypeptides. For example, an ORF containing the gene *yejL*, which has one of the highest translation efficiencies by O-30S according to ribosomal profiling data, is likely to be predominantly translated from an adjacent AUG codon preceded by a sequence predicted to pair with the ASD of O-30S (Supplementary Fig. 14). Second, studies that have advocated both 30S and 70S re-initiation suggested that the presence of the SD sequence is essential for efficient re-initiation and correct selection of downstream initiation sites^47,48^. Our results with unmodified ribosome subunits strongly suggest that longer dwell times in the bound state are linked to translation re-initiation. Hence, shorter average dwell-times in the mRNA-bound state for ASD-modified 30S subunits are in agreement with these studies. In addition, ASD-modified 30S might bind to mRNAs unproductively, and dissociate without proceeding to proper initiation. It is likely that all three factors contribute to the observed results.

On average it takes only twice longer for O-ASD ribosomes to find initiation sites compared to unmodified 30S (2.6 s+/−0.2 s vs 1.3 s+/−0.1 s, Fig. 7b). Incorporation of mutations G722C-U723A and C1192U lead to longer time needed for initiation (5.7 s+/−0.7 s and 5.6 s+/−0.2 s, respectively), probably reflecting less affinity to mRNAs. These three 30S variants, however, are likely to have similar preferences in what genes to translate, given the similar average dwell-times in the bound state (Fig. 7b). Finally, the most severely affected 30S mutant, with the additional C1400U mutation in the decoding center, shows dramatically different transition frequencies between free and mRNA-bound diffusion states, with a long-lived free state (11 s+/−1 s), interrupted only by relatively short binding events for 2.4 s+/−0.2 s (Fig. 7b). Hence, these orthogonal subunits seem not to translate endogenous mRNAs to basically any extent at all.

## Discussion

We have presented a comprehensive analysis of the diffusional behavior of *E. coli* ribosomes, and developed a toolset to study global kinetics of mRNA translation directly in living cells with a broad range of times that can be measured. Using this approach, we were able to shed light on fundamental aspects regarding the mechanism of translation initiation, not attainable from experiments in reconstituted systems, or population-averaged in vivo assays such as ribosome profiling.

Firstly, we found that both *E. coli* ribosomal subunits show indistinguishable average mRNA bound times. Hence, we see no evidence of widespread re-initiation on downstream ORFs by scanning 30S^47^. On the other hand, by comparing our estimates for the average mRNA bound time and the time required for translation of an average protein, we estimate that ≥ 50% of the 70S complexes do re-initiate in translation of polycistronic mRNAs without dissociating, in line with proposals from recent indirect experiments suggesting 70S re-initiation as a common mechanism in *E. coli*^48^. Our results from experiments with the translation initiation inhibitor AtaT further support this notion.

Secondly, previous studies along with results from the experiments with KSG presented here, strongly indicate that this antibiotic only inhibits canonical 30S initiation and does not inhibit initiation mediated by 70S.

Thirdly, by analyzing kinetics of translation elongation and initiation at different temperatures we find that the elongation rate decreases with decreasing temperature, in line with previous results. However, we also find that initiation, with respect to mRNA binding of the ribosome, occurs with comparable rates or even faster at lower temperatures.

Fourthly, we examined the importance of the SD-ASD interaction for translation initiation, and show that it has only a modest impact on the overall initiation efficiency. However, the significantly shorter mRNA-bound state dwell-times of the mutated ribosomes suggest that their function is, nevertheless, dramatically perturbed. In light of our results regarding 70S re-initiation, one likely explanation for shorter dwell-times in the bound state is that the SD-ASD interaction is crucial for 70S re-initiation on downstream ORFs, as has also been suggested previously^48^.

We do recognize that our results are, to some extent, model-dependent, particularly with respect to the diffusion threshold with which we distinguish free from mRNA-bound subunits. It should, however, be noted, that our main conclusions are based on bound-state dwell-times under different conditions, and these results are more robust than HMM estimated free-state parameters (see Supplementary Fig. 10).

Finally, in addition to mechanistic insights into prokaryotic translation, we conclude that our single-molecule tracking method allows quick evaluation of the translational state of cells, and detection of even subtle changes in state occupancies or state transition rates. After preparation of cells with labeled ribosomal subunits, this information is readily accessible within minutes without any downstream sample processing, thus minimizing the risk of experimental artifacts. The method further allows for testing of multiple conditions and to introduce changes over the course of the experiment. Hence, the method opens up new possibilities to investigate how bacteria rapidly adapt to changing conditions, such as antibiotics treatment or starvation conditions, and regulate their protein synthesis machinery for optimal resource allocation.

## Methods

### Construction of bacterial strains and growth conditions

For cloning purposes all PCRs were done using Q5 high-fidelity DNA polymerase (NEB) and Gibson Assembly was performed using NEBuilder HiFi DNA assembly master mix (NEB) according to manufacturer’s protocols. Strains expressing L1-, L9-, L25-, and S2-HaloTag were constructed using λ Red assisted recombineering^66^. First, the pKD4 plasmid (Addgene #45605) was amplified by inverse PCR using primers pKD4_F and pKD4_R (Supplementary Table 1) and Gibson-assembled with the HaloTag gene amplified with primers HaloTag_F and HaloTag_R (Supplementary Table 1) and pFA6a-HaloTag-KanMX6 (Addgene #87029) as a template. Next, the HaloTag-KmR containing fragments were amplified from the resulted pKD4-HaloTag plasmid with pairs of primers X-Halotag-F and X-Halotag-R, where X is the ribosomal protein to be mutated (Supplementary Table 1). The amplified fragments were used for recombineering to insert HaloTag-KmR cassettes in the genome of *E. coli* MG1655 at the C-terminus of each selected gene^66^.

Insertion of the MS2 aptamer into different positions of the *rrnB* operon encoded in the pAM552 plasmid (a gift from A. Mankin lab, as in Addgene #154131) was performed by inverse PCR using primers Y-MS2-F and Y-MS2-R (Supplementary Table 1), where Y designate the ribosomal helix which was modified, followed by re-circularization through PNK phosphorylation of PCR product ends and ligation by T4 ligase (NEB). Similarly, to create a plasmid for tracking of ribosomes with mutated ASD sequence, the MS2 aptamer insertion at h6 was performed by inverse PCR of the pSC101-O-ribosome (a gift from J. Chin lab) using primers h6-MS2-F and h6-MS2-R (Supplementary Table 1) and subsequent re-circularization resulting in plasmid pSC101-O-ribosome-h6-MS2. Selection of mutation C1400U in 16S rRNA in pSC101-O-ribosome-h6-MS2 was confirmed by Sanger sequencing of isolated plasmids from independent colonies showing faster growth on LB plates.

To create a plasmid in which only the ASD sequence is mutated and additional mutations (G722C, U723A, and C1192U) are not present, the fragment containing the corresponding positions was amplified using oligonucleotides Ribo600-F and Ribo1465-R (Supplementary Table 1) from the pAM552 plasmid, and the PCR product was Gibson assembled with the fragment of the plasmid pSC101-O-ribosome-h6-MS2 amplified using primers Ribo1465-F and Ribo600-R (Supplementary Table 1). The resulting plasmid pSC101-oASD-h6-MS2, when transformed in *E. coli* MG1655, caused high level of toxicity and instability. Therefore, a set of plasmids with a weaker apFAB59 promoter from the BIOFAB collection^67^ (p59) regulating expression of mutated ribosomal operon was created. First, the P1-P2 promoter present in pSC101-O-ribosome-h6-MS2 was exchanged with the p59 promoter by inverse PCR using primers P59-rrnB-F and P-rrnB-R (Supplementary Table 1), and subsequent re-circularization resulting in plasmid pSC101-P59-O-ribosome-h6-MS2. Next, a derivative of this plasmid in which only the ASD sequence is mutated was created using Gibson Assembly as explained above, yielding the plasmid pSC101-P59-oASD-h6-MS2. Finally, the plasmid pSC101-P59-oASD-C722-A723-h6-MS2, in which 16S rRNA contains the mutated ASD sequence and substitutions G722C and U723A, was created by inverse PCR using pSC101-P59-oASD-h6-MS2 as a template and primers C722-A723-F and 721-R (Supplementary Table 1), with subsequent re-circularization.

Several plasmids that allow expression of MS2CP-HaloTag were created. First, the lacUV5 promoter was inserted in the pCOLADuet vector (Novagen) by inverse PCR using primers pCOLA-lac-F and pCOLA-lac-R (Supplementary Table 1), followed by re-circularization by ligation which resulted in the pCOLA-lacUV5 plasmid. Then, the gene for single-chain tandem dimer of the high affinity version of MS2 coat protein and the gene for HaloTag were Gibson-assembled with the pCOLA-lacUV5 plasmid. Corresponding fragments were obtained by PCR amplification using pairs of primers: (i) MS2CPd-F and MS2CPd-R for amplification of MS2CP; (ii) HaloTag-F and HaloTag-R for amplification of HaloTag; (iii) pCOLA-GA-F and pCOLA-GA-R for the linearized plasmid (Supplementary Table 1). This resulted in in the pCOLA-lacUV5-MS2CP-HaloTag plasmid. Next, a fragment from the pCOLA-lacUV5-MS2CP-HaloTag plasmid, containing the *lacI* gene, lacUV5 promoter and MS2CP-HaloTag fusion, was amplified with primers MS2CP-Halo-F (Supplementary Table 1) and MS2CP-Halo-R (Supplementary Table 1). This fragment was fused via Gibson Assembly with two fragments: (i) a DNA fragment containing the p15 origin of replication and the gene for chloramphenicol acetyltransferase, and (ii) the *rrnB* terminator sequence. The first fragment was amplified using p15-Cam-F and p15-Cam-R (Supplementary Table 1) from the plasmid pEVOL-pBpF (Addgene #31190), and the second fragment was obtained from the pBAD/HisB vector (Invitrogen) by amplification using primers rrnBterm-F and rrnBterm-R (Supplementary Table 1). The resulting plasmid is called p15-lacUV5-MS2CP-HaloTag. Finally, a derivative of this plasmid in which the *lacI* gene was removed and the lacUV5 promoter was swapped with a weak constitutive promoter apFAB124 from the BIOFAB collection^67^ was created by inverse PCR using primers p124-MS2-Halo-F and p124-MS2-Halo-R (Supplementary Table 1) and subsequent re-circularization.

A plasmid pBAD30-ataT containing the gene for the AtaT toxin (a gift from S. Dubiley) from the AtaRT toxin-antitoxin system with a *P*_*BAD*_ promoter regulating the expression^55^ was modified to reduce the expression level by substituting the strong AGGAGG Shine-Dalgarno to the weak TTCTCA sequence by inverse PCR using primers AtaT-F and AtaT-weakSD-R (Supplementary Table 1) and subsequent re-circularization resulting in plasmid pBAD30-SD_weak_-AtaT.

All unique biological materials used are available from the corresponding author upon request.

### Calculation of doubling time

Bacterial strains were grown on LB agar plates overnight at 37 °C. For each tested strain, six individual colonies were grown in LB media until they reached OD_600_ = 0.1–1. The cells were then diluted to OD_600_ = 0.0001 in fresh LB and growth kinetics were recorded at 37 °C in a 96-well microplate (100 µl/well) using Spark 10 M microplate reader (Tecan) with measurements of OD_600_ performed every 5 min after 1 min shaking. After subtraction of medium background, the minimal doubling time was calculated using a custom MATLAB script, which fits growth curves of cells with an exponential function and identifies 60 min intervals of maximal growth rate for each curve. The data from six independent wells were fitted separately and the results were averaged.

### HaloTag labeling and sample preparation

*E. coli* cells containing HaloTag were grown on LB agar plates overnight at 37 °C. Several colonies were used to inoculate EZ Rich Defined Medium (RDM, Teknova) supplemented with 0.2% glucose. Cells were grown at 37 °C with shaking to OD_600_ = 0.5–1, harvested by centrifugation at 2000 x g, resuspended in 150 µl of fresh RDM media, and JF549 HaloTag ligand (a gift from Lavis lab) was added to the final concentration of 0.5 µM. After incubation with the ligand for 30 min at 25 °C, cells were washed twice using M9 media supplemented with 0.2% glucose, then incubated in RDM media at 37 °C with shaking for 60 min to facilitate the release of unbound ligand from cells. After incubation, cells were washed 3 times using M9 media supplemented with 0.2% glucose. Finally, cells were resuspended in RDM to OD_600_ ≈ 0.003 and sparsely spread onto an agarose pad prepared with RDM and 2% agarose (SeaPlaque GTG Agarose, Lonza). The sample was placed on the microscope with a cage incubator maintaining temperature at 37 ± 2 °C. Cells were grown for 120 min forming mini-colonies before image acquisition. To evaluate the effect of temperature on translation kinetics, experiments were performed at 25 ± 1 °C and 30 ± 1 °C and incubation time before imaging was 360 min and 200 min, respectively.

For KSG treatment experiments, cells were incubated on agarose pads at 37 °C for 2 h after which RDM media supplemented with 0.2% glucose and containing either 20 µg/ml or 2 mg/ml KSG was injected to the sample with growing mini-colonies. Imaging of cells was performed 60 min after injection of antibiotic. For experiment in which production of AtaT toxin was induced, *E. coli* MG1655 expressing S2- or L9-HaloTag and transformed with the pBAD30-SD_weak_-AtaT plasmid were grown on an agarose pad to form mini-colonies for 2 h at 37 °C. Production of the toxin was induced by injection of RDM media supplemented with 0.2% glucose and 0.2% arabinose. Imaging was performed 70 min later.

Each experimental dataset included 2–7 replicated microscopy experiments, each comprising 30–100 cell colonies (8–100 cells each). The data were found to be consistent in between different repetitions and were combined for analysis.

### Optical setup

An inverted microscope (Nikon Ti-E) in combination with a CFI Apo TIRF 100 × 1.49 NA objective (Nikon) was used. Bright-field and fluorescence images were acquired using an iXon 897 Ultra EMCCD camera (Andor) coupled to an additional 2.0 × lens (Diagnostic Instruments DD20NLT). Phase-contrast imaging was performed with an Infinity 2–5 M camera (Lumenera). To track JF549 bound to HaloTag, a 553 nm laser (SLIM-553, Oxxius, 150 mW) with a power density of 3 kW/cm^2^ on the sample plane was used in stroboscopic illumination mode with 3 ms laser pulses per 30 or 60 ms camera exposures. The microscope was controlled using the μManager software package. Data acquisition from multiple positions was performed using custom μManager plugins.

### Single-particle tracking and analysis of trajectories

Data analysis was performed using custom-made analysis pipelines written in MATLAB. Boundaries of individual cells were extracted from phase contrast images using a previously published algorithm^68^. Segmentation masks for incorrectly segmented cells were manually removed in order to exclude these cells from subsequent analysis. Fluorescent spots were detected using the radial symmetry-based method^69^. Refinement of spot positions and estimation of position uncertainties were performed using symmetric Gaussian spot modelling and maximum aposteriori fitting^24^. Two-dimensional trajectory building was done using uTrack^70^. Trajectories were built in segmented cells starting from the time-point when only one spot remained in the cell in current and following frames, allowing gaps with 1 missing point. Spots with width (std) > 280 nm, with amplitude < 50 photons, or outside live cells (> 3 pixels outside of boundaries) were excluded. The trajectory building was optimized to avoid detection of “unphysiological” transitions by setting the search radius for connection of dots between consecutive frames to 8 pixels (0.64 µm) and 12 pixels (0.96 µm) for experiments performed with 30 and 60 ms frame rates, correspondingly. Using a previously published HMM algorithm (uncertainSPT)^6,24^, we analyzed diffusional properties of tracked molecules by fitting all trajectories with length of ≥ 10 steps obtained for each experimental condition to a pre-set number of diffusion states. The algorithm makes explicit use of spot coordinates as well as localization uncertainties of each spot and handles missing data points^24^. Mean dwell times were calculated from the diagonal elements of the resulted HMM transition matrix, i.e., from the average probability of staying in a diffusive state, thus allowing the estimation of dwell times which are longer than the span of trajectories. To condense HMM models with many states to a coarse grained two-state model, the hidden states were classified as ‘mRNA bound’ and ‘free’ using a threshold value of 0.25 μm^2^/s. Occupancies and dwell-times presented in bar plots are all calculated as weighted mean values from model sizes 7−11, with error bars representing weighted standard deviation.

For mean square displacement (MSD) analysis, trajectories were cut into segments of 7 positions, and linear fits passing through the origin was made using the equation ${MSD}(t)=2{nD}\triangle t\cdot t$, where $t=1,2,3$ (first 3 points), $n=2$ is the dimension of the trajectories, $\triangle t=30{ms}$ is the time step, and the apparent diffusion coefficient $D$ is the fitting parameter.

### Reporting summary

Further information on research design is available in the Nature Research Reporting Summary linked to this article.

## Supplementary information

Supplementary Information

Description of Additional Supplementary Files

Supplementary Data 1-28

Supplementary Movie 1

Reporting Summary

## Data Availability

The data that support this study are available from the corresponding author upon reasonable request. Microscopy data generated and analysed during the current study are available in the SciLifeLab Data Repository, 10.17044/scilifelab.18020801. Source data are provided with this paper.

## Source data

Source Data

## Code availability

The computational code used for analysis and plotting is available in the SciLifeLab Data Repository, 10.17044/scilifelab.18020801.
