# Supplementary material for: Direct measurements of mRNA translation kinetics in living cells
Source: Nat Commun. 2022 Apr 6;13:1852. doi: 10.1038/s41467-022-29515-x (PMC8986856; doi:10.1038/s41467-022-29515-x)
Supplement: Supplementary file 1 — Supplementary Information [file 41467_2022_29515_MOESM1_ESM.pdf]

SUPPLEMENTARY INFORMATION

Direct measurements of mRNA translation kinetics in living cells

Mikhail Metelev, Erik Lundin, Ivan L Volkov, Arvid H Gynnå, Johan Elf, and Magnus Johansson*
Department of Cell and Molecular Biology, Uppsala University
*Correspondence: m.johansson@icm.uu.se

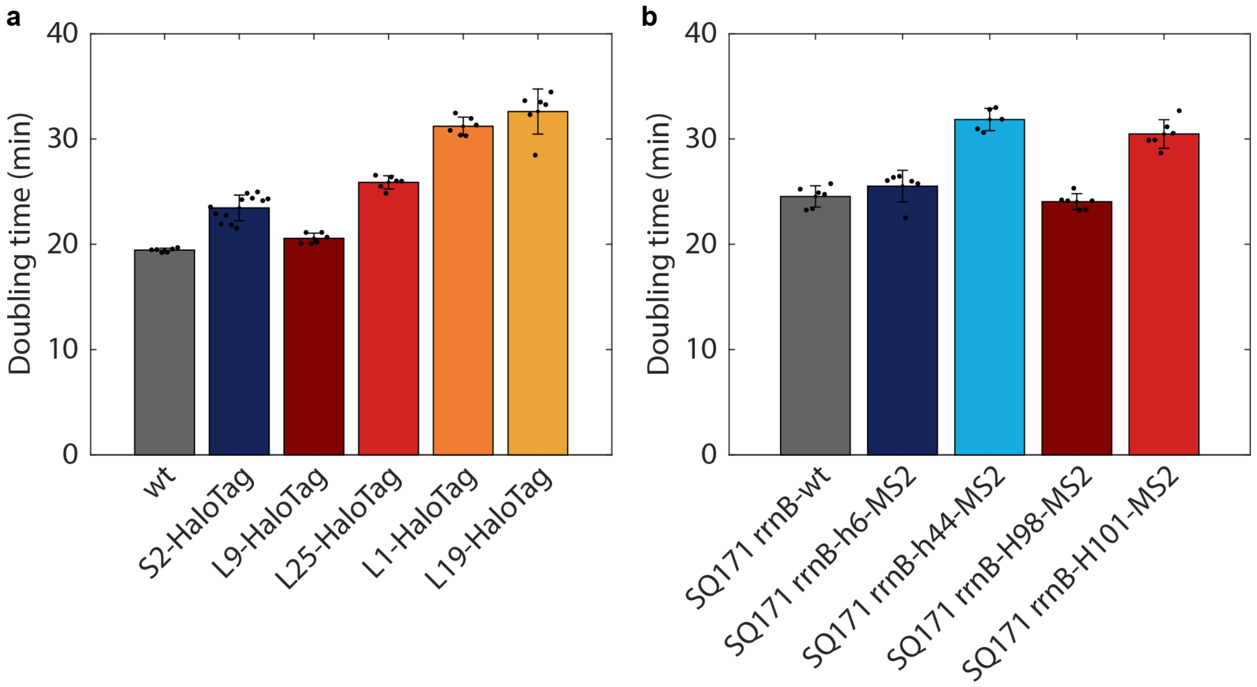

Supplementary Fig. 1. Growth of *E. coli* strains constructed for tracking of ribosomal subunits. **a**. Doubling times of *E. coli* cells expressing S2-HaloTag, L9-HaloTag, L25-HaloTag, L1-HaloTag, L19-HaloTag, and isogenic *E. coli* MG1655. Bars and error bars represent mean and standard deviation, respectively, calculated from 6 independent biological replicates (12 replicates for S2-HaloTag), where each individually measured doubling time is shown as a dot. **b**. Doubling time of *E. coli* SQ171 strain which lacks all chromosomal rRNA operons and carrying plasmid pAMM552 expressing either the wt *rrnB* operon or its mutated versions in which the MS2 aptamer was inserted in one of the helixes (helix 6, helix 44, H98 or H101). Bars and error bars represent mean and standard deviation, respectively, calculated from 6 independent biological replicates (5 replicates for rrnB-h44-MS2), where each individually measured doubling time is shown as a dot.

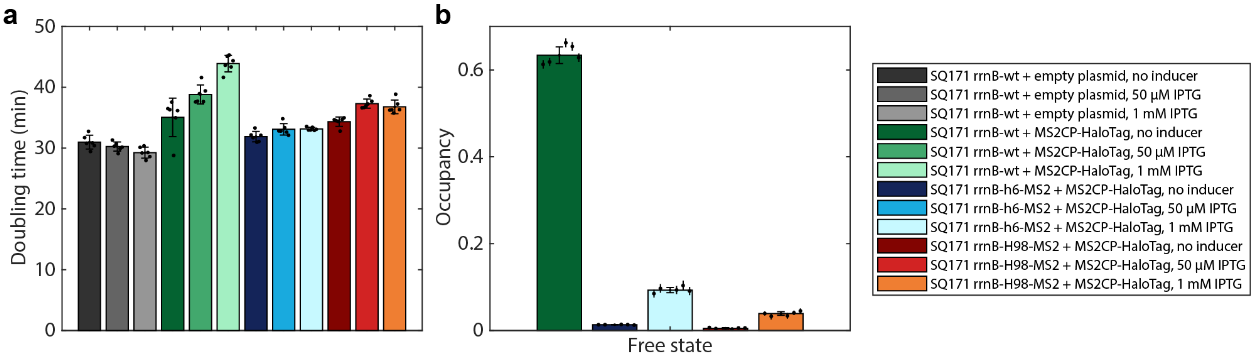

Supplementary Fig. 2. The effect of MS2CP-HaloTag production on cells expressing h6-, or H98-MS2 modified ribosomes. **a**. The effect of MS2CP-HaloTag expression on the doubling time of *E. coli* SQ171 containing either the wt *rrnB* operon or mutants with MS2 aptamer inserted in helices h6 or H98. The plasmid p15-lacUV5-MS2CP-HaloTag was used to express MS2CP-HaloTag by inducing with different concentrations of IPTG (0, 50 µM, and 1 mM). The bars and error bars represent mean and standard deviation, respectively, calculated from 6 independent biological replicates, where each individually measured doubling time is shown as a dot. **b**. HMM-estimated occupancy of MS2CP-HaloTag in the diffusional state >1.5 µm^2/s representing free proteins not bound to ribosomes. The appearance of free MS2CP-HaloTag molecules upon induction with 1 mM IPTG indicates that the level of production exceeds or is comparable with that of ribosomes. The data shows weighted averages from coarse-grained HMM-fitted model sizes 7-11, using 1.5 µm^2/s as cutoff. Error bars with caps represent weighted standard deviation, calculated from individual model sizes (7-11) with bootstrap estimated standard errors (shown as error bars without caps). n = 56046, 53404, 35817, 126411, and 53107 trajectory steps collected from 2, 2, 2, 2, and 2 independent experiments for dark green (SQ171 rrnB-wt + MS2CP-HaloTag, no inducer), dark blue (SQ171 rrnB-h6-MS2 + MS2CP-HaloTag, no inducer), light blue (SQ171 rrnB-h6-MS2 + MS2CP-HaloTag, 1 mM IPTG), dark red (SQ171 rrnB-H98-MS2 + MS2CP-HaloTag, no inducer), and orange (SQ171 rrnB-H98-MS2 + MS2CP-HaloTag, 1 mM IPTG), respectively. Source data are provided as a Source Data file.

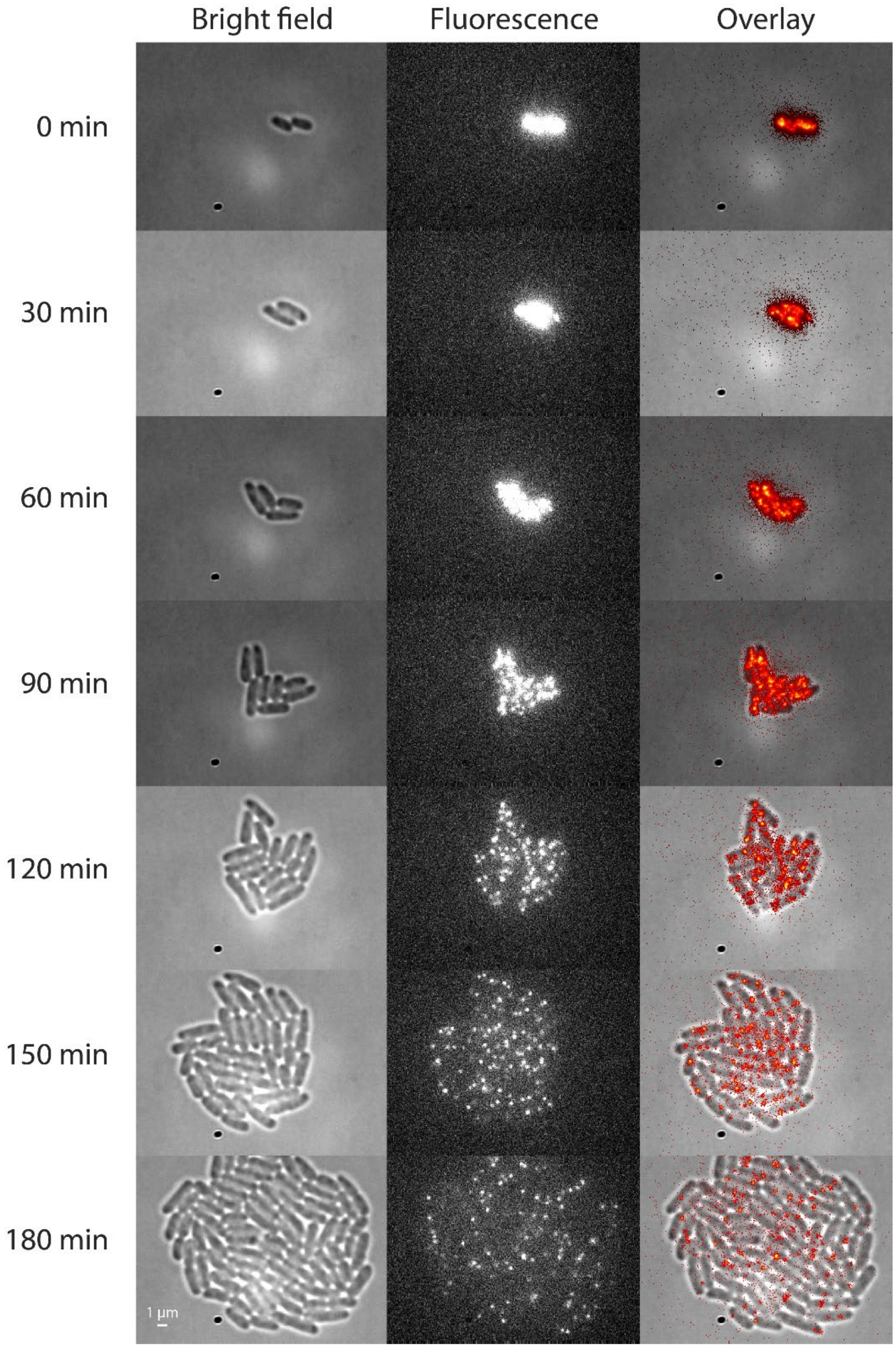

Supplementary Fig. 3. Bright-field and fluorescence images (553 nm laser illumination) of L9-HaloTag expressing *E. coli* cells after labeling with the JF549 HaloTag ligand. Similar results were observed for all microscopy experiments included in the study.

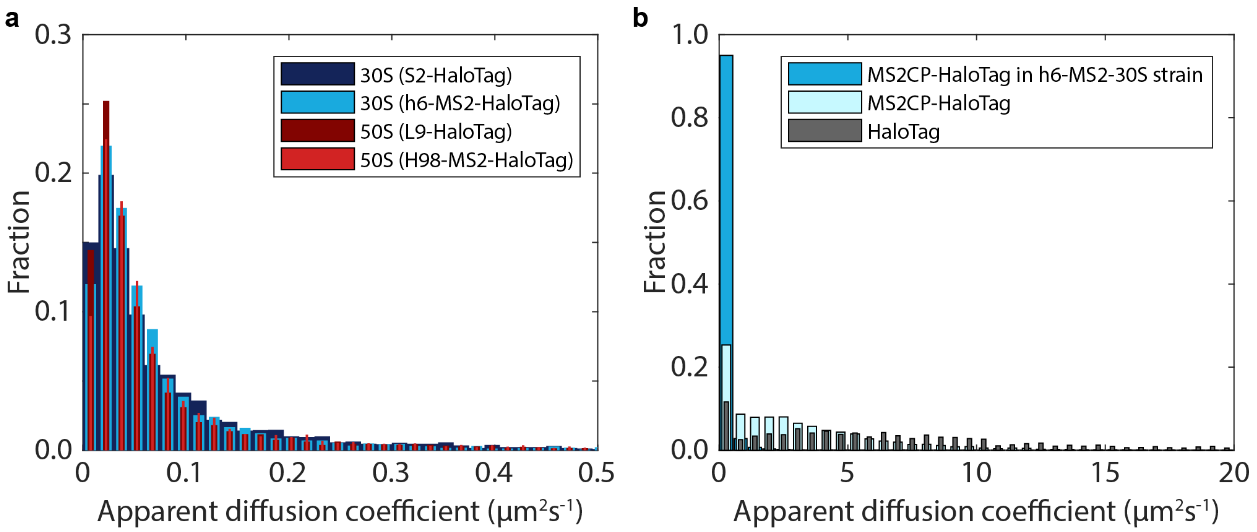

Supplementary Fig. 4. Apparent diffusion coefficients estimated from mean-squared-displacement. **a**. Distribution of apparent diffusion coefficient for 30S or 50S HaloTag labeled ribosomal subunits. **b**. Distribution of apparent diffusion coefficient for free HaloTag and the MS2CP-HaloTag fusion in cells lacking the MS2 RNA aptamer. Data for diffusion of the MS2CP-HaloTag fusion in cells with the MS2 RNA aptamer inserted into a subpopulation of the 16S rRNA is shown for comparison. The average diffusion coefficient for HaloTag and the MS2CP-HaloTag were calculated to be 12 $\mu m^2/s$ and 4 $\mu m^2/s$, respectively. The apparent diffusion coefficients in both panels were estimated from mean-squared-displacement analysis of diffusion trajectory segments of 7 frames. Fluorescence data were acquired at 30 ms frames for panel **a**, and 5 ms for panel **b**.

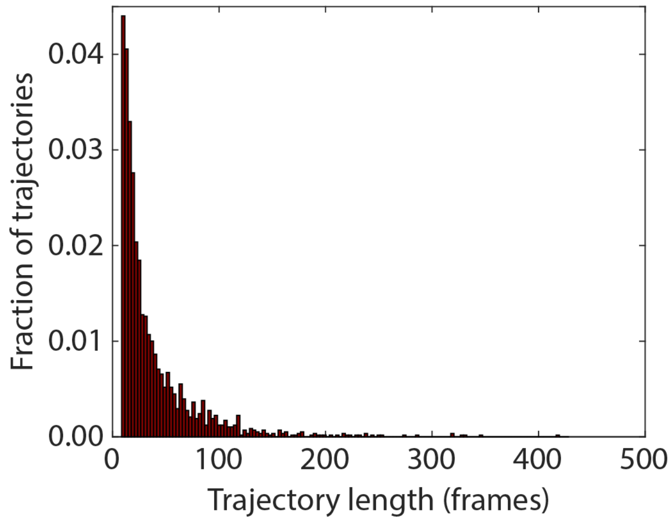

Supplementary Fig. 5. Distribution of trajectory lengths from a single 50S (L9-HaloTag) tracking experiment. Only trajectories of length 10 frames or longer were included in the subsequent HMM analysis.

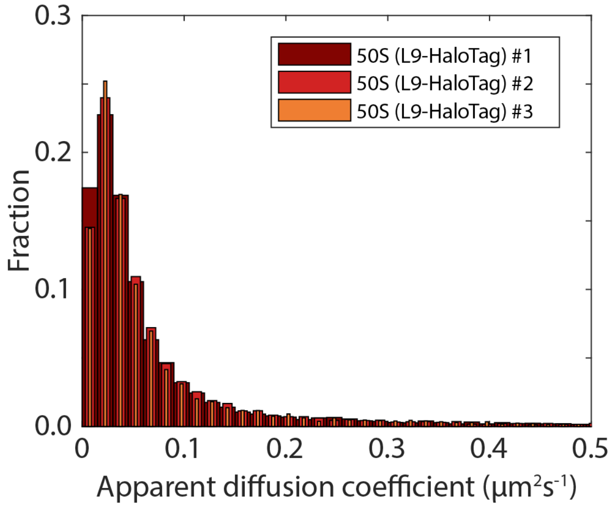

Supplementary Fig. 6. Distribution of apparent diffusion coefficient for L9-HaloTag labeled 50S ribosomal subunits, estimated from mean-squared-displacement analysis of diffusion trajectory segments of 7 frames from three separate experiments.

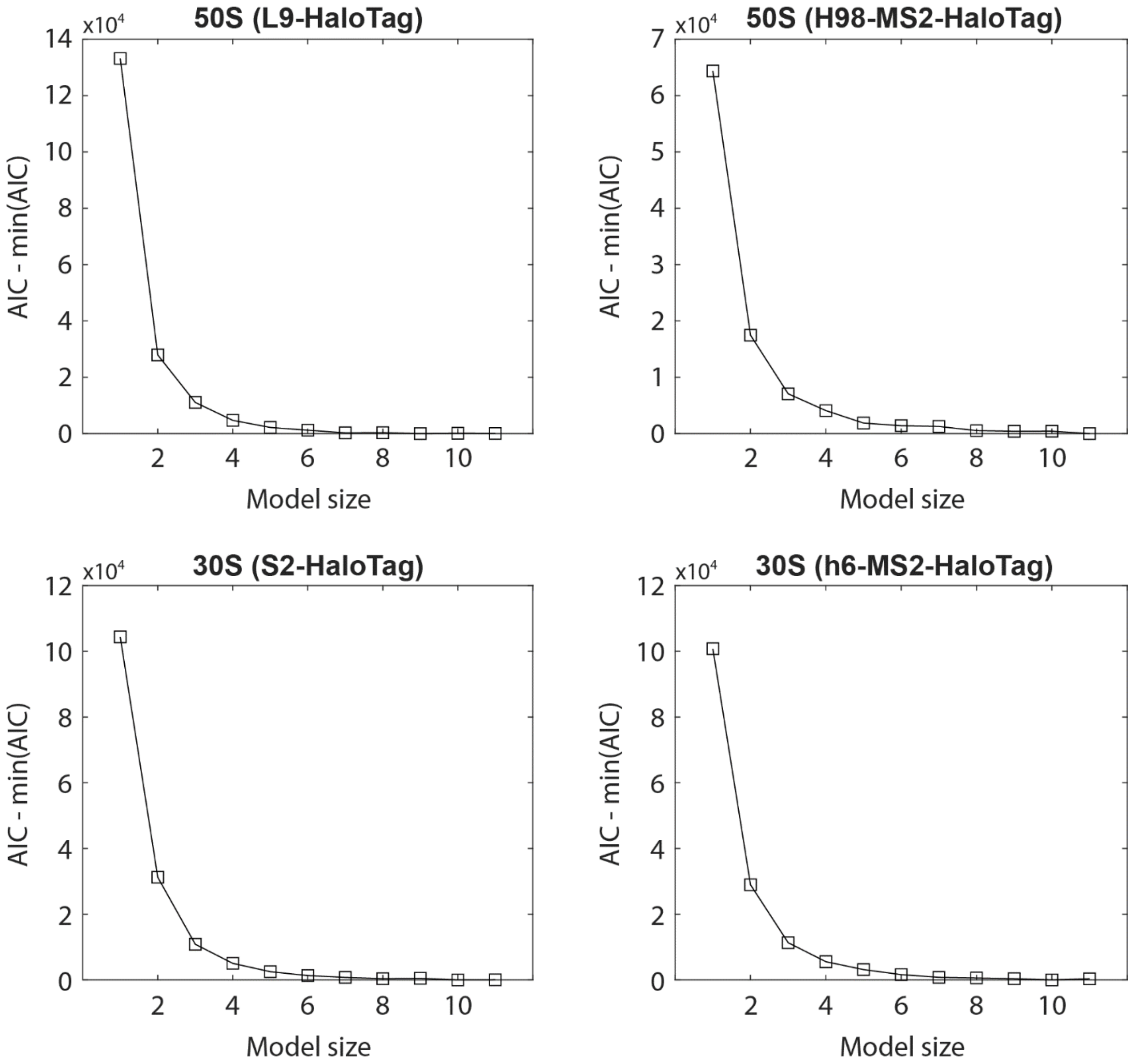

Supplementary Fig. 7. AIC values, relative to the lowest, for HMM fitted models with different number of diffusive states.

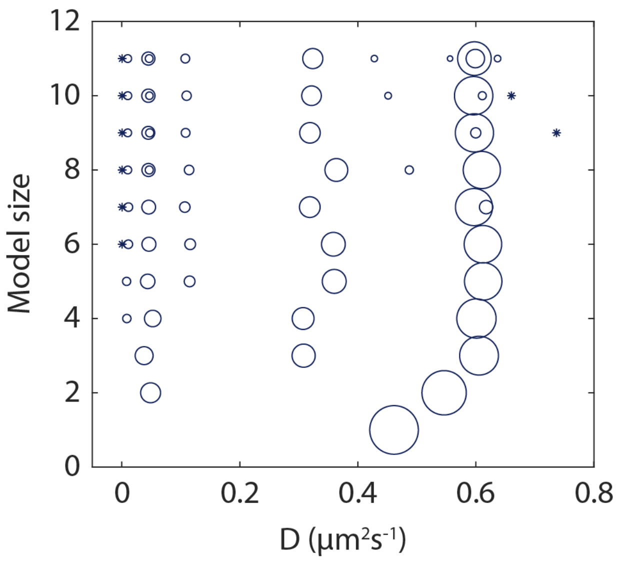

Supplementary Fig. 8. HMM-estimated diffusion state occupancies for labelled O-30S-U1400 subunits. The area of the circles is proportional to the relative occupancy. Occupancies lower than 1% are shown as *. All models are also shown in Supplementary Data 9.

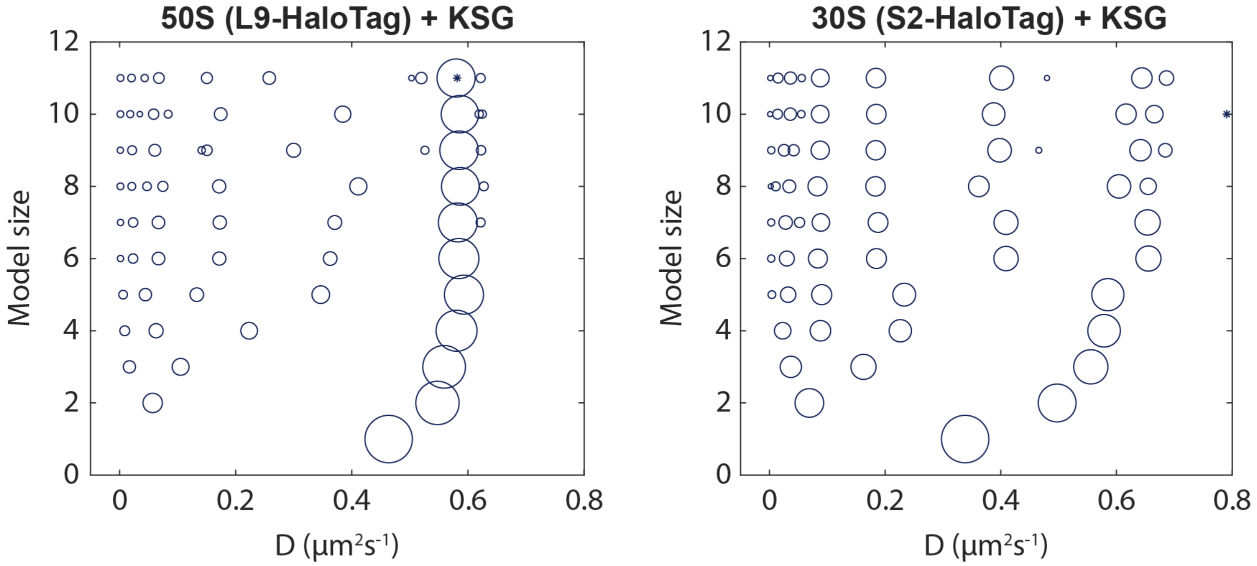

Supplementary Fig. 9. HMM-estimated diffusion state occupancies for labelled 50S and 30S subunits in the presence of 2 mg/ml KSG. The area of the circles is proportional to the relative occupancy. Occupancies lower than 1% are shown as *. All models are also shown in Supplementary Data 10-11.

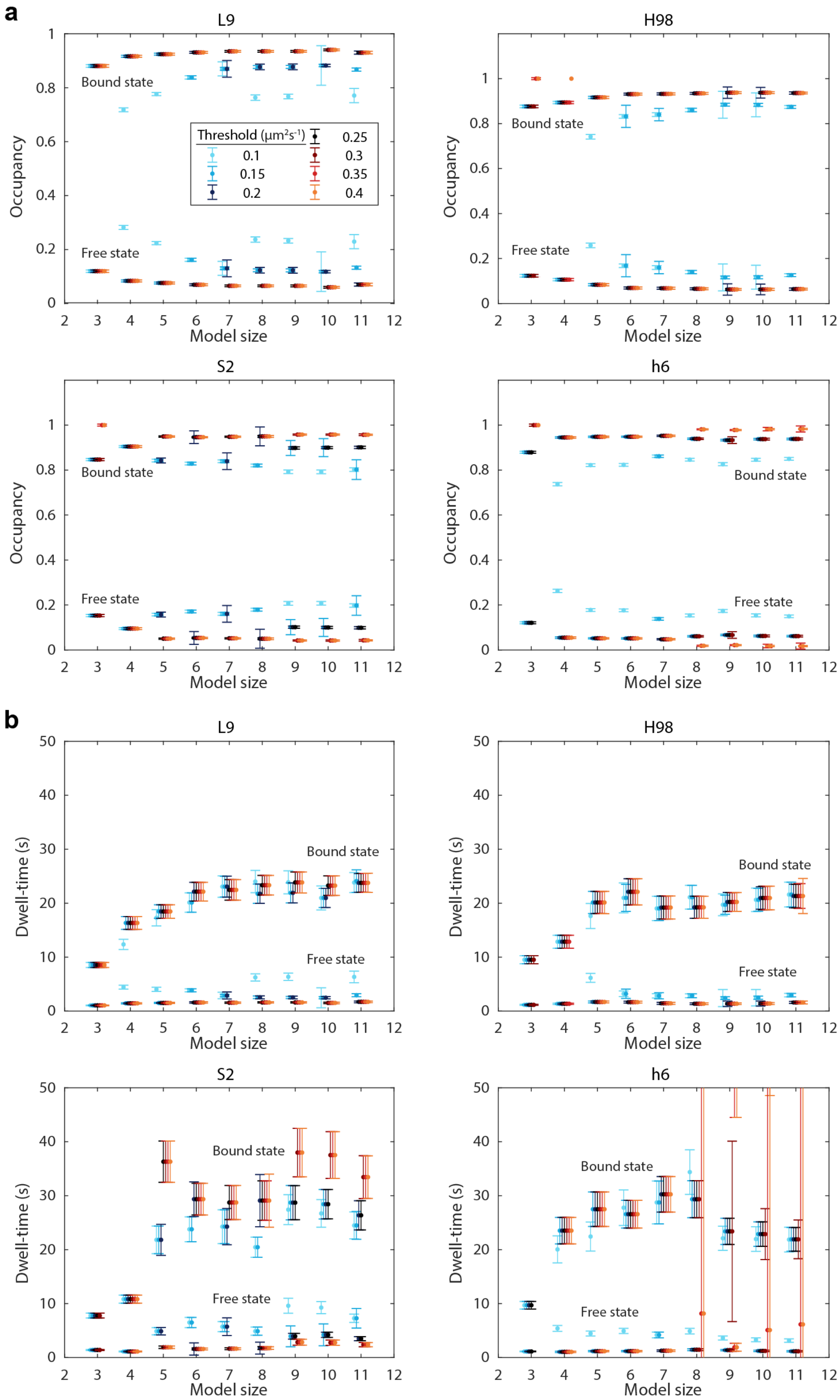

Supplementary Fig. 10. Estimated occupancy (**a**) and dwell-time (**b**) of ribosomal subunits in the mRNA-bound state or the freely diffusive state for different HMM fitting model sizes and for different coarse-graining thresholds. Error bars represent bootstrap estimated standard errors. All models are also

available in Supplementary Data 13. n = 179569, 115349, 161623, and 191950 trajectory steps collected from 3, 3, 7, and 6 independent experiments for L9, H98, S2, and h6 labelling, respectively. Source data are provided as a Source Data file.

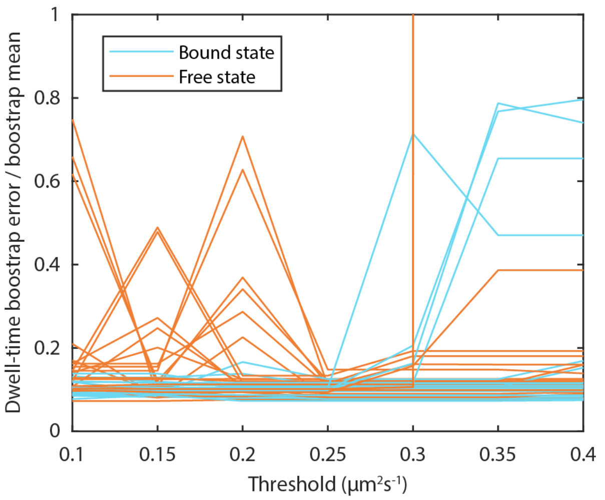

Supplementary Fig. 11. Bootstrap estimated standard error of dwell-times, at different coarse-graining thresholds, for L9-, S2-, h6-, and H98-labelled ribosome subunits, using HMM model sizes 6-11, normalized to the respective bootstrap estimated mean.

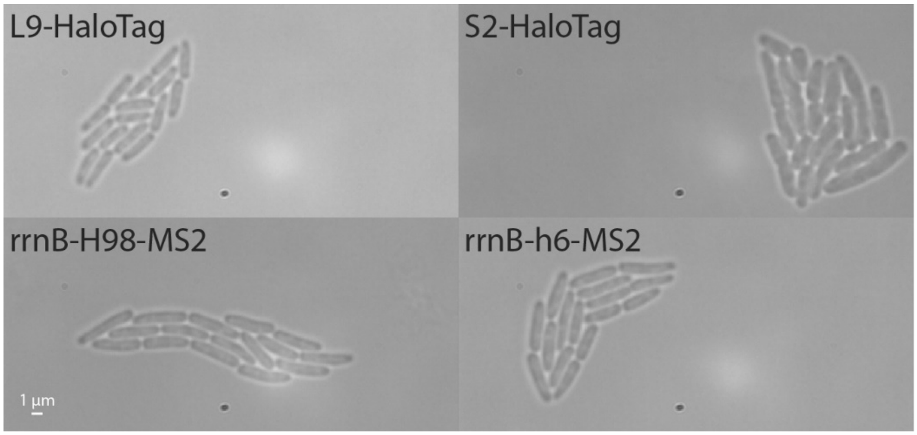

Supplementary Fig. 12. Bright-field microscopy images of cell strains used for ribosome subunit labelling. Mini-colonies of *E. coli* MG1655 cells expressing either S2-HaloTag, L9-HaloTag, or co-expressing MS2CP-HaloTag with *rrnB* mutated versions in which the MS2 aptamer was inserted in helix 6 and H98. Images are representative for all 3, 3, 7, and 6 independent experiments for L9, H98, S2, and h6 labelling, respectively.

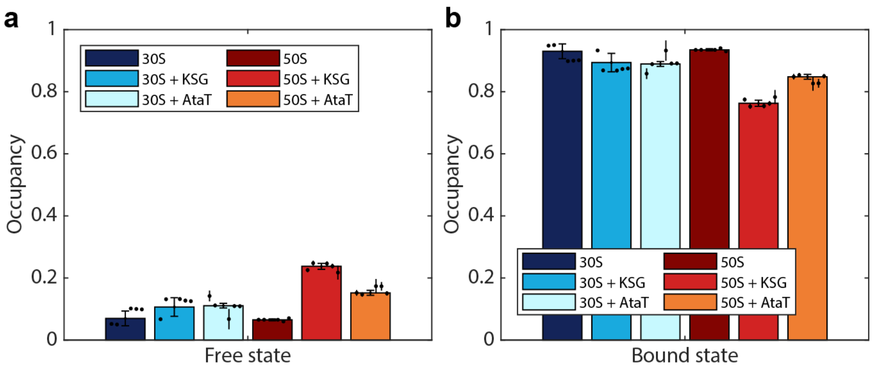

Supplementary Fig. 13. HMM-estimated occupancy of 30S and 50S subunits in the freely diffusing state (**a**) or in the mRNA bound state (**b**) in presence or absence of 20 µg/ml KSG or during the expression of the toxin AtaT. The data shows weighted averages from coarse-grained HMM-fitted model sizes 7-11 (Supplementary Data 12). Results for individual model sizes (2-11) are shown in Supplementary Data 4, 6, 16-19. Error bars with caps represent weighted standard deviation, calculated from individual model sizes (7-11) with bootstrap estimated standard errors (shown as error bars without caps). n = 161623, 141242, 101518, 179569, 182163, and 103391 trajectory steps collected from 7, 2, 5, 3, 2, and 5 independent experiments for 30S, 30S + KSG, 30S + AtaT, 50S, 50S + KSG, and 50S + AtaT, respectively. Source data are provided as a Source Data file.

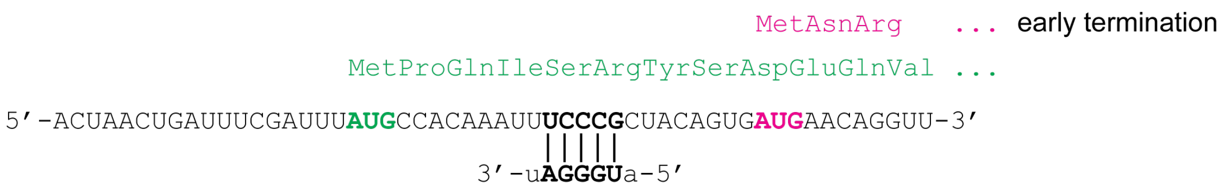

Supplementary Fig. 14. The ribosomal binding site of the *yejL* mRNA. The annotated AUG start codon and beginning of the normally translated protein sequence are shown in green color. The sequence upstream of AUG in magenta color is predicted to pair with the ASD of O-30S (interaction is shown in bold) and is located at the optimal distance for efficient translation initiation. Translation initiation from the alternative (magenta) AUG results in frame-shift and early termination.

Supplementary Table 1. Oligonucleotides used for cloning.

Name	Sequence, 5'-3'
HaloTag-F	acggctgacatgggaattagatggcagaaatcggtactgg
HaloTag-R	gatattcatatggaccatggctagccggaaatctcgagcgtcgacagc
pKD4-F	cgctcgagatttccggctagccatggtccatatgaatatcctccttagt
pKD4-R	ccagtaccgatttctgccatctaattcccatgtcagccgttaagtg
L1-Halotag-F	gttgcagttgaccaggctggcctgagcgcttctgtaaacggcgcagaaatcggtactggc
L1-Halotag-R	gggtaagattgtagacaaaatcaccgcccacgtaaaggcagtgtaggctggagctgcttc
L9-Halotag-F	gaagtattcgcgaaagtgatcgtaaacgtagtagctgaaggcgcagaaatcggtactggc
L9-Halotag-R	accaatggtcggcgtttttacgtctcgttgaataacgaaagtgtaggctggagctgcttc
L19-Halotag-F	cgtactggtaaggctgctcgtatcaaagagcgtcttaacggcgcagaaatcggtactggc
L19-Halotag-R	ggccagcccttcttaacaggatgtcgcttaagcgaaatcagtgtaggctggagctgcttc
L25-Halotag-F	tacaaaccgaagctgcagcacatcgacttcgttcgcgctggcgcagaaatcggtactggc
L25-Halotag-R	accccgccggagcggggtttttacaacttattcagcaaagtgtaggctggagctgcttc
S2-Halotag-F	ctggcttcccaggcggaagaaagcttcgtagaagctgagggcgcagaaatcggtactggc
S2-Halotag-R	tctgcaactcgaactattttgggggagttatcaagccttagtgtaggctggagctgcttc
h6-MS2-F	actagttttgatgaggatcacccatctttactagtcttctttgctgacgagtggcg
h6-MS2-R	cttcttcctgttaccgttcgacttg
h44-MS2-F	actagttttgatgaggatcacccatctttactagtggagggcgcttaccactttgtg
h44-MS2-R	ggttaagctacctacttcttttgc
H98-MS2-F	actagttttgatgaggatcacccatctttactagtagggtcctgaaggaacgttga
H98-MS2-R	agggtcagggagaactcatctcgg
H101-MS2-F	actagttttgatgaggatcacccatctttactagttgcgttgagctaaccggtac
H101-MS2-R	tgcgcttacacacccggccta
Ribo1465-F	ccactttgtgattcatgactggggtgaag
Ribo1456-R	cttcaccccagtcatgaatcacaaagtgg
Ribo600-F	gatgtgaaatccccgggctcaacctgggaactgcatctgatact
Ribo600-R	tcagatgcagttcccaggttgagcccggggat
P59-rrnB-F	ttgacaattaatcatccggctcgatacttacagccatcccgcgccgctgagaaaaagcga
P-rrnB-R	gaggaaatttaaaataattttctgaccgcg
C722-A723-F	caggcgaaggcggccccctgga
721-R	cggtattcctccagatctctacgc
pCOLA-lac-F	gtggaattgtgagcggataacaatttgctgccaccgctgagcaataa
pCOLA-lac-R	acattatacgagccggaagcataaagtgtaaagcatttcctaatgcaggagtcgcataag
MS2CPd-F	ttgtgagcggataacaatttctagcaggaggaattgatggcttct
MS2CPd-R	aagccagtaccgatttctgcgcgcagaagggggatccgtaga
HaloTag-F	tctacggatcccccttctgcgcgcagaaatcggtactggctttcc
HaloTag-R	tattgctcagcggtggcagcctagccggaaatctcgagcg
pCOLA-GA-F	cgctcgagatttccggctaggctgccaccgctgagc
pCOLA-GA-R	ccatcaattcctcctgctagaaattgttatccgctcacaattccac
lacUV5-MS2CP-Halo-F	taagcttggtacccaaccaagctcactgcccgctttcc
lacUV5-MS2CP-Halo-R	caaaacagccaagcaagcttctagccggaaatctcgagcg
rrnBterm-F	cgctcgagatttccggctagaagcttgcttggctgttttgg
rrnBterm-R	agcaggcttttcgaatttggaacaaaagagtttgtagaaacgcaaaaagg
p15-Cam-F	tttctacaaactcttttgttccaaattcgaaaagcctgctcaac
P15-Cam-R	ctggaaagcgggcagtgagcttggttgggtaccaagcttatcgatga
p124-MS2-Halo-F	gaattcaaaagatctaaagaggagaaaggatctatggcttctaactttactcagttcgtt
p124-MS2-Halo-R	atggctgtaagtattcgccgcaagggataaatgtcgagctgtcaaacatgagaattacaa
AtaT-F	atggatgatctgacgatagagattc
AtaT-weakSD-R	ttttaattgagaatgaattcgctagcccaaaaaaacg
