# Supplementary material for: Direct measurements of mRNA translation kinetics in living cells
Source: Nat Commun. 2022 Apr 6;13:1852. doi: 10.1038/s41467-022-29515-x (PMC8986856; doi:10.1038/s41467-022-29515-x)
Supplement: Supplementary file 2 — Description of Additional Supplementary Files [file 41467_2022_29515_MOESM2_ESM.pdf]

Description of Additional Supplementary Files

File Name: Supplementary Data 1
Description: Results of HMM analysis for tracking of L9-HaloTag for replicate #1

File Name: Supplementary Data 2
Description: Results of HMM analysis for tracking of L9-HaloTag for replicate #2

File Name: Supplementary Data 3
Description: Results of HMM analysis for tracking of L9-HaloTag for replicate #3

File Name: Supplementary Data 4
Description: Results of HMM analysis for tracking of L9-HaloTag

File Name: Supplementary Data 5
Description: Results of HMM analysis for tracking of H98-MS2-HaloTag

File Name: Supplementary Data 6
Description: Results of HMM analysis for tracking of S2-HaloTag

File Name: Supplementary Data 7
Description: Results of HMM analysis for tracking of h6-MS2-HaloTag

File Name: Supplementary Data 8
Description: Results of HMM analysis for tracking of O-30S

File Name: Supplementary Data 9
Description: Results of HMM analysis for tracking of O-30S-U1400

File Name: Supplementary Data 10
Description: Results of HMM analysis for tracking of L9-HaloTag in cells treated with KSG 2000 µg/ml

File Name: Supplementary Data 11
Description: Results of HMM analysis for tracking of S2-HaloTag in cells treated with KSG 2000 µg/ml

File Name: Supplementary Data 12
Description: Weighted mean of model size 7 - 11

File Name: Supplementary Data 13
Description: Coarse graining with different thresholds

File Name: Supplementary Data 14
Description: Average protein length estimation based on proteomics data

File Name: Supplementary Data 15
Description: Average protein length estimation based on ribosome profiling data

File Name: Supplementary Data 16
Description: Results of HMM analysis for tracking of L9-HaloTag in cells expressing AtaT toxin

File Name: Supplementary Data 17
Description: Results of HMM analysis for tracking of L9-HaloTag in cells treated with KSG 20 µg/ml

File Name: Supplementary Data 18
Description: Results of HMM analysis for tracking of S2-HaloTag in cells expressing AtaT toxin

File Name: Supplementary Data 19
Description: Results of HMM analysis for tracking of S2-HaloTag in cells treated with KSG 20 µg/ml

File Name: Supplementary Data 20
Description: Results of HMM analysis for tracking of L9-HaloTag in cells growing at 25°C (30 ms frame time)

File Name: Supplementary Data 21
Description: Results of HMM analysis for tracking of L9-HaloTag in cells growing at 30°C (30 ms frame time)

File Name: Supplementary Data 22
Description: Results of HMM analysis for tracking of L9-HaloTag in cells growing at 25°C (60 ms frame time)

File Name: Supplementary Data 23
Description: Results of HMM analysis for tracking of L9-HaloTag in cells growing at 30°C (60 ms frame time)

File Name: Supplementary Data 24
Description: Results of HMM analysis for tracking of L9-HaloTag in cells growing at 37°C (60 ms frame time)

File Name: Supplementary Data 25
Description: Results of HMM analysis for tracking of O-ASD-30S, expression of the operon from the promoter apFAB59

File Name: Supplementary Data 26
Description: Results of HMM analysis for tracking of O-ASD-C722-A723, expression of the operon from the promoter apFAB59

File Name: Supplementary Data 27
Description: Results of HMM analysis for tracking of O-30S, expression of the operon from the promoter apFAB59

File Name: Supplementary Data 28
Description: Results of HMM analysis for tracking of O-30S-U1400, expression of the operon from the promoter apFAB59

File Name: Supplementary Movie 1
Description: Fluorescence microscopy data of L9-HaloTag labeled 50S ribosomal subunits, acquired with 3 ms laser exposures per 30 ms camera frame. Playback is 33 frames per second (i.e. real-time). The left panel shows raw data, whereas the right panel shows the cell outlines (segmented from phase-contrast images) as well as automatically detected diffusion trajectories, colorcoded with respect to coarse-grained diffusion states estimated from HMM analysis. Diffusion trajectories were built in cells from the time-point where only one spot per cell remained in the current and all subsequent frames.
