# Supplementary material for: Direct measurements of mRNA translation kinetics in living cells
Source: Nat Commun. 2022 Apr 6;13:1852. doi: 10.1038/s41467-022-29515-x (PMC8986856; doi:10.1038/s41467-022-29515-x)
Supplement: Supplementary file 5 — Reporting Summary [file 41467_2022_29515_MOESM5_ESM.pdf]

nature portfolio

Corresponding author(s): Magnus Johansson

Last updated by author(s): Mar 10, 2022

Reporting Summary

Nature Portfolio wishes to improve the reproducibility of the work that we publish. This form provides structure for consistency and transparency in reporting. For further information on Nature Portfolio policies, see our Editorial Policies and the Editorial Policy Checklist.

Statistics

For all statistical analyses, confirm that the following items are present in the figure legend, table legend, main text, or Methods section.

n/a	Confirmed	
☐	☒	The exact sample size (*n*) for each experimental group/condition, given as a discrete number and unit of measurement
☐	☒	A statement on whether measurements were taken from distinct samples or whether the same sample was measured repeatedly
☒	☐	The statistical test(s) used AND whether they are one- or two-sided<br>*Only common tests should be described solely by name; describe more complex techniques in the Methods section.*
☒	☐	A description of all covariates tested
☒	☐	A description of any assumptions or corrections, such as tests of normality and adjustment for multiple comparisons
☐	☒	A full description of the statistical parameters including central tendency (e.g. means) or other basic estimates (e.g. regression coefficient) AND variation (e.g. standard deviation) or associated estimates of uncertainty (e.g. confidence intervals)
☒	☐	For null hypothesis testing, the test statistic (e.g. *F*, *t*, *r*) with confidence intervals, effect sizes, degrees of freedom and *P* value noted<br>*Give P values as exact values whenever suitable.*
☒	☐	For Bayesian analysis, information on the choice of priors and Markov chain Monte Carlo settings
☒	☐	For hierarchical and complex designs, identification of the appropriate level for tests and full reporting of outcomes
☒	☐	Estimates of effect sizes (e.g. Cohen's *d*, Pearson's *r*), indicating how they were calculated

Our web collection on statistics for biologists contains articles on many of the points above.

Software and code

Policy information about availability of computer code

Data collection	Automated data acquisition was performed using µManager (1.4.23), and an in-house written µManager plugin.
Data analysis	MATLAB R2018a (MathWorks), uncertainSPT 0.9.2 (github.com/bmelinden/uncertainSPT). The different parts of the image analysis pipeline, i.e., cell segmentation, spot detection, etc. were handled through custom written MATLAB scripts. All data and code for analysis and plotting is available in the SciLifeLab Data Repository, https://doi.org/10.17044/scilifelab.18020801.

For manuscripts utilizing custom algorithms or software that are central to the research but not yet described in published literature, software must be made available to editors and reviewers. We strongly encourage code deposition in a community repository (e.g. GitHub). See the Nature Portfolio guidelines for submitting code & software for further information.

Data

Policy information about availability of data

All manuscripts must include a data availability statement. This statement should provide the following information, where applicable:

- Accession codes, unique identifiers, or web links for publicly available datasets
- A description of any restrictions on data availability
- For clinical datasets or third party data, please ensure that the statement adheres to our policy

Microscopy data generated and analysed during the current study is available in the SciLifeLab Data Repository, https://doi.org/10.17044/scilifelab.18020801.

Field-specific reporting

Please select the one below that is the best fit for your research. If you are not sure, read the appropriate sections before making your selection.

☒ Life sciences ☐ Behavioural & social sciences ☐ Ecological, evolutionary & environmental sciences

For a reference copy of the document with all sections, see nature.com/documents/nr-reporting-summary-flat.pdf

Life sciences study design

All studies must disclose on these points even when the disclosure is negative.

Sample size	Each experimental dataset included 2–7 replicated microscopy experiments, each comprising 30–100 cell colonies (8-100 cells each). The data from several repetitions were combined for analysis. The sample size for analyzed data is denoted in total number of trajectory steps (n) and specified for each dataset. Based on analyses of single-molecule microscopy simulations (Fig. 5 in Volkov et al. (2018) NatChemBiol (https://doi.org/10.1038/s41589-018-0063-y)), we estimate that at least 30000 trajectory steps are needed to reach convergence in the dwell-time estimation of the current experiments. This is fulfilled in all cumulated datasets. The number of trajectory steps needed, 30000, however, depends on the dynamics of the system and is related to the number of state transitions occurring per time unit. Since this was not known beforehand, and since the number of trajectory steps extracted from a single experiment varies dramatically, the number of experimental repetitions could not be predetermined and hence varies between the different datasets. No data from already performed experiments were excluded from the analysis, despite reaching 30000 trajectory steps with less repetitions included.
Data exclusions	Fluorescence data from bacterial cells not completely covered in the field of view (i.e. half cells) or outside laser illumination, and incorrectly segmented cells (with deviation from cell outline based on visual inspection) were omitted from the analysis. Fluorescence movies of colonies with no or very few fluorescent molecules (≤ 2 fluorophores per cell colony) were not included in the analysis. Fluorescence movies of colonies with apparent x-y movement of the sample during data acquisition were not included in the analysis. On a few occasions (≤ 10 cells per dataset, <1 % of cells), cells with what appeared to be fluorescent impurities or unspecifically bound fluorophores (e.g. aggregates of fluorophores), were omitted from the analysis. All these exclusion criteria, except for the x-y movement, were pre-established based on previous studies (Volkov et al. (2018) NatChemBiol (https://doi.org/10.1038/s41589-018-0063-y). We did not expect x-y movement of the sample during acquisition, however, so this criterium was established during data processing.
Replication	Each microscopy experiment was performed in 2–7 replicas using different bacterial colonies obtained from the same bacterial stock. The data were found consistent in between different repetitions (see e.g. Supplementary Data 1-3) and were combined for Hidden Markov Model analysis (as described above under “Sample size”).
Randomization	No experimental groups were used. Not relevant.
Blinding	The experimental setup was the same for all types of experiments, and the analysis of experimental data was performed automatically with the same parameters used in all sets of experiments. Hence, blinding is not relevant.

Reporting for specific materials, systems and methods

We require information from authors about some types of materials, experimental systems and methods used in many studies. Here, indicate whether each material, system or method listed is relevant to your study. If you are not sure if a list item applies to your research, read the appropriate section before selecting a response.

Materials & experimental systems

n/a	Involved in the study	
☒	☐	Antibodies
☒	☐	Eukaryotic cell lines
☒	☐	Palaeontology and archaeology
☒	☐	Animals and other organisms
☒	☐	Human research participants
☒	☐	Clinical data
☒	☐	Dual use research of concern

Methods

n/a	Involved in the study	
☒	☐	ChIP-seq
☒	☐	Flow cytometry
☒	☐	MRI-based neuroimaging
